# Exemplar scoring identifies genetically separable phenotypes of lithium responsive bipolar disorder

**DOI:** 10.1038/s41398-020-01148-y

**Published:** 2021-01-11

**Authors:** Abraham Nunes, William Stone, Raffaella Ardau, Anne Berghöfer, Alberto Bocchetta, Caterina Chillotti, Valeria Deiana, Franziska Degenhardt, Andreas J. Forstner, Julie S. Garnham, Eva Grof, Tomas Hajek, Mirko Manchia, Manuel Mattheisen, Francis McMahon, Bruno Müller-Oerlinghausen, Markus M. Nöthen, Marco Pinna, Claudia Pisanu, Claire O’Donovan, Marcella D. C. Rietschel, Guy Rouleau, Thomas Schulze, Giovanni Severino, Claire M. Slaney, Alessio Squassina, Aleksandra Suwalska, Gustavo Turecki, Rudolf Uher, Petr Zvolsky, Pablo Cervantes, Maria del Zompo, Paul Grof, Janusz Rybakowski, Leonardo Tondo, Thomas Trappenberg, Martin Alda

**Affiliations:** 1 Department of Psychiatry, Dalhousie University, Halifax, NS Canada; 2 Faculty of Computer Science, Dalhousie University, Halifax, NS Canada; 3 Unit of Clinical Pharmacology & San Giovanni di Dio Hospital, University Hospital of Cagliari, Cagliari, Italy; 4 Charité University Medical Center, Campus Charité Mitte, Institute for Social Medicine, Epidemiology and Health Economics, Berlin, Germany; 5 Department of Biomedical Sciences, Section of Neuroscience and Clinical Pharmacology, University of Cagliari, Cagliari, Italy; 6 Institute of Human Genetics, University of Bonn, School of Medicine & University Hospital Bonn, Bonn, Germany; 7 Centre for Human Genetics, University of Marburg, Marburg, Germany; 8 Department of Biomedicine, University of Basel, Basel, Switzerland; 9 Mood Disorders Center of Ottawa, Ottawa, Ontario Canada; 10 Department of Psychiatry, University of Toronto, Toronto, Ontario Canada; 11 Department of Medical Sciences and Public Health, Section of Psychiatry, University of Cagliari, Cagliari, Italy; 12 Department of Pharmacology, Dalhousie University, Halifax, Nova Scotia Canada; 13 Department of Psychiatry, University of Wurzburg, Wurzburg, Germany; 14 National Institute of Mental Health, Bethesda, MD USA; 15 Charité Universitätsmedizin-Berlin, Berlin, Germany; 16 Centro Lucio Bini, Cagliari e Roma, Cagliari, Italy; 17 Central Institute of Mental Health, Medical Faculty Mannheim, Heidelberg University, Mannheim, Baden-Württemberg Germany; 18 Montreal Neurological Institute, McGill University, Montreal, QC Canada; 19 Institute of Psychiatric Phenomics and Genomics, Munich, Germany; 20 Department of Adult Psychiatry, Poznan University of Medical Sciences, Poznan, Poland; 21 Department of Mental Health, Poznan University of Medical Sciences, Poznan, Poland; 22 Department of Psychiatry, McGill University Health Centre, Montreal, Québec Canada; 23 Department of Psychiatry, Charles University, Prague, Czech Republic; 24 Department of Psychiatric Nursing, Poznan University of Medical Sciences, Poznan, Poland; 25 Harvard Medical School and McLean Hospital, Boston, MA USA

**Keywords:** Predictive markers, Personalized medicine

## Abstract

Predicting lithium response (LiR) in bipolar disorder (BD) may inform treatment planning, but phenotypic heterogeneity complicates discovery of genomic markers. We hypothesized that patients with “exemplary phenotypes”—those whose clinical features are reliably associated with LiR and non-response (LiNR)—are more genetically separable than those with less exemplary phenotypes. Using clinical data collected from people with BD (*n* = 1266 across 7 centers; 34.7% responders), we computed a “clinical exemplar score,” which measures the degree to which a subject’s clinical phenotype is reliably predictive of LiR/LiNR. For patients whose genotypes were available (*n* = 321), we evaluated whether a subgroup of responders/non-responders with the top 25% of clinical exemplar scores (the “best clinical exemplars”) were more accurately classified based on genetic data, compared to a subgroup with the lowest 25% of clinical exemplar scores (the “poor clinical exemplars”). On average, the best clinical exemplars of LiR had a later illness onset, completely episodic clinical course, absence of rapid cycling and psychosis, and few psychiatric comorbidities. The best clinical exemplars of LiR and LiNR were genetically separable with an area under the receiver operating characteristic curve of 0.88 (IQR [0.83, 0.98]), compared to 0.66 [0.61, 0.80] (*p* = 0.0032) among poor clinical exemplars. Variants in the Alzheimer’s amyloid–secretase pathway, along with G-protein-coupled receptor, muscarinic acetylcholine, and histamine H1R signaling pathways were informative predictors. This study must be replicated on larger samples and extended to predict response to other mood stabilizers.

## Introduction

Bipolar disorder (BD) is a lifelong illness characterized by recurrent manias, depressions, and a relatively high suicide risk^1,2^. Initiation of mood stabilizers, of which lithium is a first-line option^3^, occurs approximately a decade after symptom onset on average^4^, and the trial-and-error process of pharmacological optimization for BD may lengthen this time. However, by predicting patients’ mood-stabilizer response, this burden of untreated illness may be reduced.

Clinical phenotypes---patient features that may be obtained by clinical interview alone---are currently the best predictors of lithium response^5^, but genomic markers likely exist. Responders often have a “classical phenotype” characterized by a completely episodic course with full inter-episode remissions, absence of rapid cycling and psychosis (particularly if mood-incongruent), and family history of fully remitting BD or lithium response in a first degree relative^6,7^. The familial nature of lithium responsive BD has been a particular motivator for the pursuit of strong genomic markers of lithium response, and polygenic scores for major depression and schizophrenia are inversely associated with lithium responsiveness^8,9^. However, predictors based on variation measured by single nucleotide polymorphisms (SNPs) remain elusive^10^.

The significant clinical heterogeneity of BD may limit the power of genomic prediction of lithium response^10^. Indeed, we have found that the clinical predictors of lithium responsiveness may differ across centers in multi-site studies^5^. However, some clinical phenotypes may consistently predict lithium responsiveness across all centers; patients with such phenotypes are the *best clinical exemplars* of lithium response and non-response, respectively. Conversely, *poor clinical exemplars* are characterized by clinical profiles that do not consistently predict lithium response or non-response. We hypothesize that biological differences will be greatest among samples of lithium responders and non-responders whose phenotypes are clinically exemplary.

The present work has two steps. First, by using a large set of clinical data on lithium-treated patients with BD collected across seven international specialist clinics, our present work develops a measure called *clinical exemplar scoring* which identifies those subjects whose lithium responsiveness can be reliably predicted from their clinical phenotype (the “best clinical exemplars” of lithium response and non-response, respectively). This method also identifies “poor clinical exemplars” of lithium response and non-response, respectively. Poor clinical exemplars are patients whose lithium responsiveness is not reliably predicted from their clinical profiles. We hypothesized that the clinical differences between the best clinical exemplars of lithium response and non-response would be reflective of factors previously associated with the “classical” bipolar phenotype.

The second step of our study leveraged the fact that genomic data were available for some subjects whose *clinical* exemplar scores were computed based on clinical features. The genomic and clinical data are not combined, but rather, we test whether genomic classification of responders vs. non-responders improves when the subject sample is restricted to only the best clinical exemplars of lithium response and non-response, respectively. We hypothesized that lithium responsiveness would be better discriminated among the best clinical exemplars, rather than the poor clinical exemplars.

## Methods

Our analysis has two steps, using two separate datasets. Step 1 uses a multi-center database of clinical variables to derive a score that identifies subjects whose clinical phenotypes reliably predict lithium response/non-response. A subset of subjects for whom we computed clinical exemplar scores in Step 1 (a North American outbred sample from Dalhousie University, Canada) also had genomic data available from participation in an entirely separate study through the Consortium on Lithium Genetics (ConLiGen). In Step 2, we evaluate whether lithium responsiveness can be better discriminated based on genomic data when the subject group is restricted only to those individuals who are the best clinical exemplars of the lithium responsive and non-responsive phenotypes, respectively. It bears repeating that absolutely no genomic data from Step 2 were included in the clinical exemplar scoring procedure of Step 1, and no clinical variables from Step 1 were included in the genomic classification procedure of Step 2. Clinical and genetic data were collected with informed consent in the context of protocols approved by the Ethics Committee of the former Health Agency of Cagliari (for Cagliari University and Centro Bini samples), and the research ethics boards of the Nova Scotia Health Authority, the McGill University Health Centre, the Royal Ottawa Hospital, and the University of Poznan.

### Step 1: Scoring and characterization of clinical exemplars

**Fig. 1:**
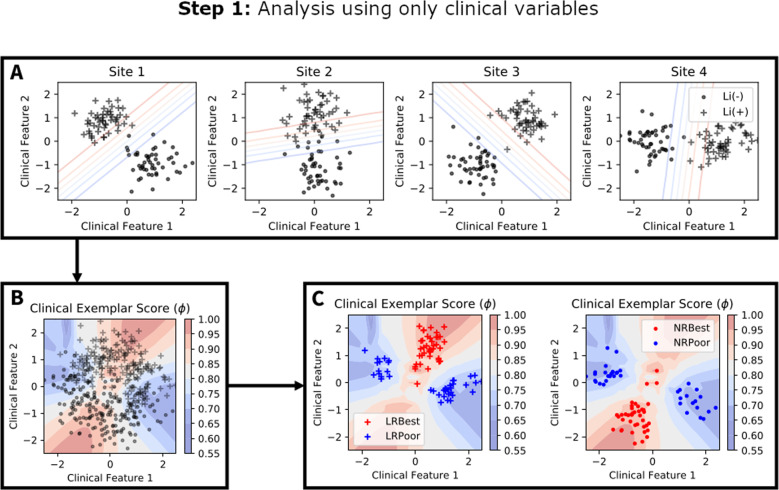
Hypothetical illustration of the clinical exemplar scoring analysis. Note that this step of the analysis is performed using the clinical feature dataset alone, and therefore has no overlap in the feature space with genomic data used in Step 2. **A** Demonstration of heterogeneity in the relationship between lithium responsiveness (depicted as “Li(+)” for responders and “Li(−)” for non-responders) and clinical features across four hypothetical sites. Overall (**A**) demonstrates how a classifier trained on different sites’ data may yield different discriminative functions. **B** Points demonstrate the aggregated dataset (“+” and “−” are responders and non-responders, respectively). Contours demonstrate regions of clinical feature space in which site-level classifiers from (**A**) agree with high accuracy on the predicted class (i.e., where they overlap). A clinical exemplar score can be computed for each subject in the clinical dataset by (1) holding his data out of the training set, (2) predicting his lithium responsiveness using site-level classifiers trained on the remaining subjects, then (3) using the site-wise prediction results to compute the clinical exemplar score. **C** Stratification of the clinical dataset according to lithium responsiveness and clinical exemplar score quartile. The “LRBest” and “NRBest” exemplars are those responders and non-responders with clinical exemplar scores above the 75^th^ percentile, respectively. The “LRPoor” and “NRPoor” exemplars are those responders and non-responders with clinical exemplar scores below the 25^th^ percentile, respectively. This stratification can be used to evaluate the clinical features that differentiate good from poor clinical exemplars of lithium response and non-response, respectively.

Figure 1 illustrates Step 1 of the present study, wherein we identify and characterize clinical phenotypes that are reliably predictive of lithium responsiveness using the *clinical exemplar score*. This step of our study uses only the clinical database, without any reference to the genomic data used for Step 2 of the analysis.

#### Clinical data collection

Clinical data collection procedures were described in Nunes et al.^5^. Data consisted of 180 variables recorded prior to instituting lithium maintenance therapy in 1266 people with BD across 7 centers (minimum treatment duration of 1 year). Datasets are described in Supplementary Table 1. Lithium response was defined as a score of ≥7 on the previously validated Alda scale^11^, which accounts for observation of appropriate lithium levels and compliance.

#### Computation of the clinical exemplar score

The best clinical exemplars should be classified accurately by models trained on data from any given site. Our overall clinical exemplar scoring protocol thus involves (A) obtaining out-of-sample predictions of every subject’s class based on models trained on each individual site’s data, then (B) summarizing accuracy and between-site agreement into a single value known as the clinical exemplar score. Figure 1 provides a visual intuition for the clinical exemplar score computation. Full technical description is provided in the supplementary materials.

We employed a random forest classifier (RFC)^12^ under the same specifications as in Nunes et al.^5^ (100 estimators; SciKit Learn implementation^13^). Similar to that study, missing data were marginalized by sampling from uninformative priors on respective variables’ domains, class imbalance was addressed using Synthetic Minority Oversampling Technique (SMOTE) with a Tomek link function. Briefly, SMOTE augments the minority class in a training set with synthetic but similar observations. The Tomek link removes synthetic observations that overlap in feature space with the majority class, or are close to a decision boundary^14^. Furthermore, neither hyperparameter optimization nor further feature set restrictions were undertaken, since hyperparameter optimization does not improve the clinical prediction model^5^ and feature informativeness is taken into account during RFC training.

For each site in the clinical predictors dataset, our predict every subject out (PESO) analysis protocol begins with a leave-one-out cross-validation run to obtain out-of-sample predictions for each of that site’s constituent subjects. We then train an RFC on that site’s data and predict lithium response in all other sites’ subjects. Each subject thus obtains one prediction of his or her response for each site’s classifier. Accuracy of each site’s prediction was calculated as the reciprocal of the absolute error for each subject.

Using the distribution of accuracies recorded for each subject, the clinical exemplar score is computed as an index of both (A) how accurately the subject’s response is predicted, and (B) the agreement across sites regarding the subject’s class. Poor clinical exemplars will score near zero (poor accuracy and poor agreement across sites). Conversely, the best clinical exemplars score near 1 (accurate classification with high agreement across sites). Subjects classified accurately with poor agreement (or vice versa) have intermediate clinical exemplar scores.

#### Comparing characteristics of the best and poor clinical exemplars in the clinical dataset

Univariate clinical feature differences were compared between the best clinical exemplars of lithium response and non-response (“LRBest” and “NRBest,” respectively; the top 25% of clinical exemplar scores per class), and the corresponding poor clinical exemplars (“LRPoor” and “NRPoor,” respectively; the bottom 25% of clinical exemplar scores per class). Continuous variables were compared using the two-sample permutation test and categorical variables were compared using the randomization chi-square test (with 10,000 replications). The significance threshold was adjusted for 116 comparisons: *α*_*c*_ = 0.05/116 = 0.0004.

### Step 2: Biological validation through genomic classification

**Fig. 2:**
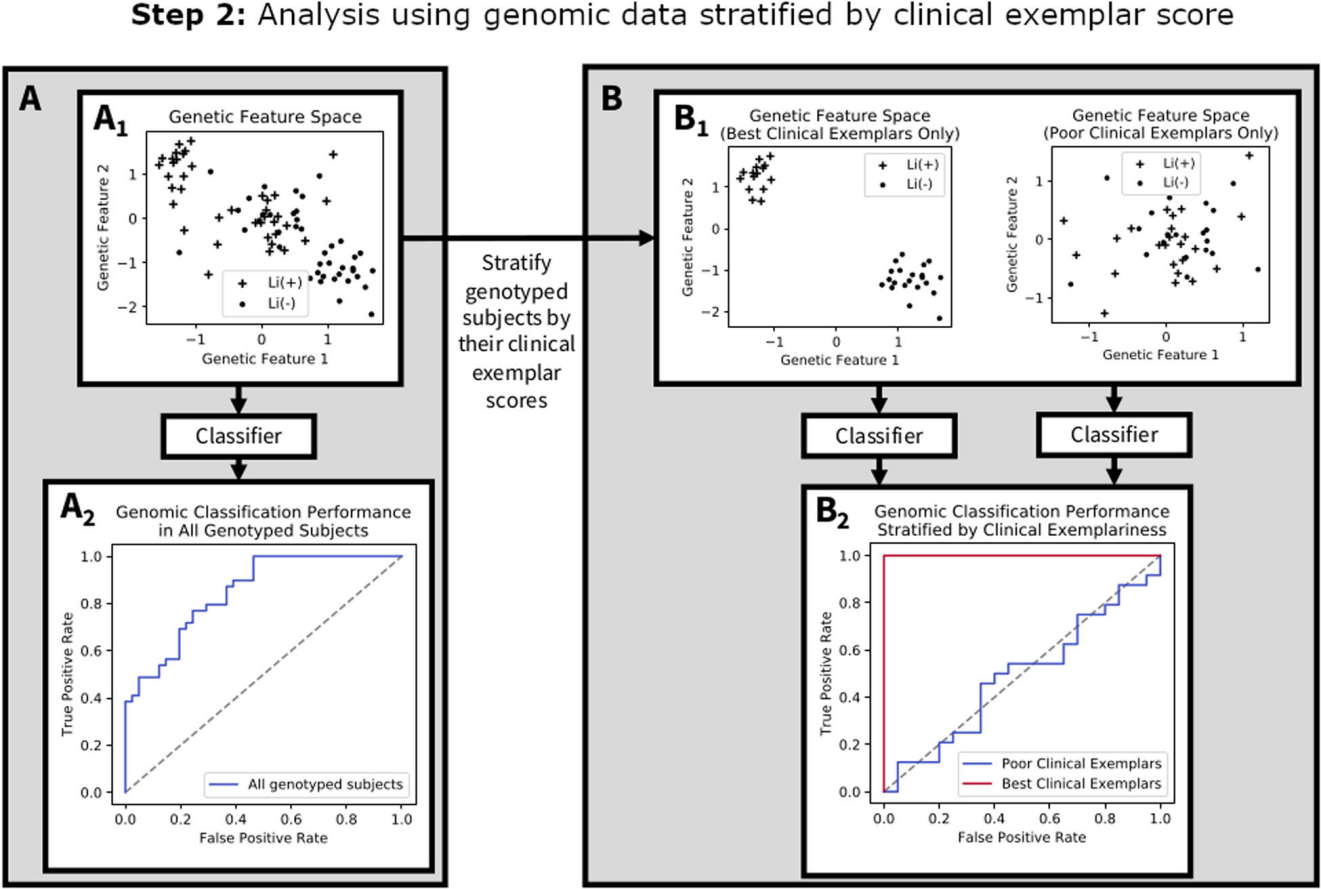
Hypothetical illustration of Step 2 of this study’s analysis, which evaluates the degree to which stratification of the subject group into best and poor clinical exemplars can improve genomic classification performance. **A** Subjects’ genotypes lie on a genotypic feature space (shown in (**A**_**1**_) as a simplified 2 dimensional plane), (**A**_**2**_) shows a hypothetical ROC curve for these aggregated data. **B** Each genotyped subject has a clinical exemplar score computed from Step 1 of the present study. The exemplar score merely identifies the degree to which a subject’s *clinical* profile (i.e., symptoms, family history, comorbidities, etc.) is reliably predictive of lithium responsiveness across sites in the *clinical dataset*; it merely allows us to define the different groups for the genomic classification analysis (best and poor clinical exemplars); (**B**_**1**_) illustrates stratification of the aggregated genotyped sample into the “Best” clinical exemplars (subjects with top 25% of clinical exemplar scores within each of the responder and non-responder groups, respectively), and the “Poor” clinical exemplars (those with the lowest 25% of clinical exemplar scores in each responsiveness class). Importantly, the clinical exemplar score is not computed based on any genomic information, nor are any variables from the clinical dataset included in the genomic classification analysis. We then apply classifiers to the genomic data in each of these best and poor clinical exemplar strata, respectively, and compare classification performance (**B**_**2**_). The hypothetical receiver operating characteristic curve in (**B**_**2**_) reflects our hypothesis, that genetic classification of lithium response will be superior among the subgroup of best clinical exemplars.

Figure 2 illustrates Step 2 of the present study, wherein we compare the genetic prediction of lithium response when genotyped subjects are stratified by their clinical exemplar scores. Recall that the stratification into “best clinical exemplars” and “poor clinical exemplars” is done exclusively in Step 1 using clinical variables, and therefore absolutely no clinical features are included in the genomic classification analysis. This step of our study uses genomic data from a relatively ethnically homogeneous subset of subjects in the ConLiGen GWAS^10^ (those sourced from Halifax, Nova Scotia, Canada) who also had detailed clinical information collected for Step 1 of the present study.

#### Genomic data collection

Genomic data, obtained as part of the ConLiGen GWAS^10^, were available for 321 of the 1266 subjects whose clinical data were analyzed in Step 1 of our study. In the ConLiGen sample, these subjects were a relatively ethnically homogeneous subset contributed by the Canadian group, which also has a similar number of responders and non-responders (i.e., “class balance”, with 159 [49.5%] responders; Supplementary Table 2). Supplementary Figs. 3 and 4 show that there was no population stratification in our subsample. We restricted the data to only the 47,465 directly genotyped SNPs for which complete data were available across all ConLiGen sites. Preprocessing and quality control were done according to Hou et al.^10^ (see Supplementary Materials).

#### Genomic classification with stratification by clinical exemplar score

For genotyped subjects, we compared the performance of a classifier applied to genomic data from (A) all 321 subject’s, (B) the poor clinical exemplars’, and (C) the best clinical exemplars’. We employed L2-penalized logistic regression (C = 1 set a priori, since this imposes a consistent prior on model weights, facilitating their comparison). Model criticism was performed under stratified-10-fold cross-validation. In the Supplementary Materials, we provide an alternative protocol using repeated shuffle-split cross-validation, in which we also test the sensitivity of our results to test set size.

Our primary outcome was the average cross-validated Matthews correlation coefficient (MCC). Classification performance differences were compared between conditions using the Kruskal-Wallis test. Where a statistically significant difference was observed (at *α* = 0.05), pair-wise comparisons were done with the Mann-Whitney U tests (at threshold *α*_*c*_ = 0.05/3 = 0.017). We secondarily report accuracy, area under the receiver operating characteristic curve (ROC-AUC), Cohen’s kappa, sensitivity, specificity, positive predictive value (PPV), and negative predictive value (NPV).

#### Gene enrichment analysis

In the model trained on the best clinical exemplars, we indexed variants whose logistic regression coefficients agreed in sign across all cross-validation folds, then applied the statistical enrichment test to the nearest associated genes using the PANTHER classification system v. 14.1^15^. For comparison, we repeated this analysis using logistic regression coefficients from the poor exemplar group. Our significance threshold was *α*_FDR_*=*0.05 where FDR indicates correction for false discovery rate. Further enrichment analysis details are provided in the Supplementary Methods.

## Results

### Step 1: Scoring and characterization of clinical exemplars

#### Heterogeneity of classification performance across sites in the predict every subject out analysis

Supplementary Fig. 5 shows that the distributions of accuracy between site-level models were variable in shape and modality, highlighting the heterogeneity in between-site classification behavior whose redress is the central motivating factor of our present study.

#### Characteristics of the best and poor clinical exemplars in the clinical dataset

Within the clinical dataset of Step 1, there were 110 individuals in LRBest and LRPoor groups, and 207 individuals in the NRBest and NRPoor groups (Table 1). Note that the groups compared in Table 1 are not those for whom we attempted genomic classification in Step 2 of the present study (those comparisons are presented in Supplementary Table 2). The LRBest group came predominantly from IGSLi (53.6%) and Ontario (21.8%), and most NRBest subjects were from Maritimes (72.5%) and Montreal (25.1%).

**Table 1:** Characteristics of the best (upper 25% of exemplar scores) and poor (lower 25% of exemplar scores) exemplars of lithium response and non-response, respectively.

	Best exemplars			Poor exemplars		
	Non-responder	Responder	*p*	Non-responder	Responder	*p*
*n*	207	110		207	110	
Male (%)	76 (36.7)	46 (41.8)	0.39826	79 (38.2)	34 (30.9)	0.21488
Age (y)	42.44 [32.12, 51.80]	54.19 [42.38, 65.46]	<0.00001	45.92 [36.39, 57.80]	59.74 [44.35, 66.12]	<0.00001
Center (%)			—			0.0001
CU	4 (1.9)	21 (19.1)		74 (35.7)	1 (0.9)	
CB	1 (0.5)	0 (0.0)		70 (33.8)	11 (10.0)	
IGSLi	0 ((0.0)	59 (53.6)		0 (0.0)	8 (7.3)	
MAR	150 (72.5)	6 (5.5)		38 (18.4)	16 (14.5)	
MTL	52 (25.1)	0 (0.0)		11 (5.3)	2 (1.8)	
ON	0 (0.0)	24 (21.8)		6 (2.9)	21 (19.1)	
POZ	0 (0.0)	0 (0.0)		8 (3.9)	51 (46.4)	
Diagnosis (%)			0.12369			0.0472
BD I	139 (67.1)	71 (64.5)		136 (65.7)	66 (60.0)	
BD II	62 (30.0)	33 (30.0)		51 (24.6)	36 (32.7)	
MDD recurrent	0 (0.0)	3 (2.7)		3 (1.4)	4 (3.6)	
MDD single				0 (0.0)	1 (0.9)	
SZA	6 (2.9)	3 (2.7)		17 (8.2)	3 (2.7)	
Age of onset (y)	19.00 [16.00, 24.00]	28.00 [21.00, 36.00]	<0.00001	22.50 [18.00, 32.25]	27.50 [18.25, 35.00]	0.16618
Onset of depression (y)	20.00 [16.00, 25.00]	30.00 [23.00, 37.00]	<0.00001	28.00 [20.00, 38.00]	30.00 [20.50, 37.50]	0.77534
Onset of mania (y)	25.00 [21.00, 32.00]	30.00 [26.00, 40.00]	0.00012	29.33 [22.00, 36.50]	32.00 [28.00, 39.75]	0.00928
Onset of hypomania (y)	26.50 [21.00, 38.50]	38.00 [25.50, 45.50]	0.00293	32.49 (14.59)	38.13 (12.16)	0.06037
Polarity episode 1 (%)			0.0002			0.011
Biphasic (D-M)	4 (2.0)	5 (5.8)		3 (5.8)	1 (2.4)	
Biphasic (M-D)	13 (6.6)	4 (4.7)		2 (3.8)	2 (4.8)	
Hypomania	19 (9.7)	8 (9.3)		10 (19.2)	3 (7.1)	
Major depression	142 (72.4)	42 (48.8)		20 (38.5)	30 (71.4)	
Mania	13 (6.6)	16 (18.6)		16 (30.8)	4 (9.5)	
Minor depression	5 (2.6)	11 (12.8)		1 ((1.9)	2 (4.8)	
Clinical course (%)			0.0001			0.0001
Chronic	14 (6.8)	0 (0.0)		8 (4.1)	21 (25.3)	
Chronic deteriorating	2 (1.0)	0 (0.0)		3 (1.5)	2 (2.4)	
Chronic fluctuating	90 (43.5)	0 (0.0)		11 (5.6)	34 (41.0)	
Completely episodic	7 (3.4)	27 (100.0)		146 (74.1)	15 (18.1)	
Continuous cycling	1 (0.5)	0 (0.0)		7 (3.6)	2 (2.4)	
Episodic with residual	93 (44.9)	0 (0.0)		22 (11.2)	9 (10.8)	
Lifetime manias, *n*	3.00 [1.00, 7.00]	2.00 [0.00, 3.00]	0.00001	3.00 [1.00, 6.00]	2.00 [1.00, 3.00]	0.02059
Lifetime depressions, *n*	5.00 [3.00, 15.00]	3.00 [2.00, 6.00]	<0.00001	4.00 [2.00, 8.00]	4.00 [2.00, 6.00]	0.03006
Lifetime mixed, *n*	0.00 [0.00, 1.00]	0.00 [0.00, 0.00]	<0.00001	0.00 [0.00, 0.00]	0.00 [0.00, 0.00]	0.40342
Lifetime multiphasic, *n*	0.00 [0.00, 1.00]	0.00 [0.00, 2.00]	0.00006	0.00 [0.00, 0.00]	0.00 [0.00, 0.00]	0.18415
Total lifetime episodes, *n*	9.00 [5.00, 24.50]	6.00 [5.00, 10.00]	<0.00001	8.00 [5.00, 15.00]	5.00 [4.00, 9.00]	0.00464
Rapid cycling (%)			0.0001			0.70113
Never	92 (47.2)	59 (98.3)		56 (93.3)	80 (96.4)	
Only on antidepressants	7 (3.6)	0 (0.0)		2 (3.3)	1 (1.2)	
Spontaneous	96 (49.2)	1 (1.7)		2 (3.3)	2 (2.4)	
Rapid mood switching (%)	47 (63.5)	0 (0.0)	0.06059	6 (21.4)	1 (1.8)	0.0049
Lifetime psychosis (%)			0.0001			0.0018
In episodes, congruent	83 (42.8)	5 (16.7)		51 (38.9)	15 (20.0)	
In episodes, incongruent	36 (18.6)	0 (0.0)		8 (6.1)	1 (1.3)	
Never	72 (37.1)	25 (83.3)		70 (53.4)	59 (78.7)	
Outside of mood episodes	3 (1.5)	0 (0.0)		2 (1.5)	0 (0.0)	
GAF at last assessment	70.00 [55.00, 75.00]	90.00 [90.00, 95.00]	<0.00001	75.00 [60.00, 86.25]	87.50 [80.00, 90.00]	0.01251
Li total score	2.00 [0.00, 4.00]	8.00 [8.00, 10.00]	<0.00001	3.00 [1.00, 5.00]	8.00 [7.00, 9.00]	<0.00001
Episodes on Li, *n*	4.00 [1.25, 10.00]	0.00 [0.00, 1.75]	0.01189	2.00 [1.00, 4.00]	1.00 [0.00, 1.50]	0.00008
Episodes pre-Li, *n*	4.00 [3.00, 12.00]	5.00 [4.00, 15.75]	0.14411	4.00 [3.00, 7.00]	4.00 [3.00, 6.00]	0.77478
SA, *n*	0.00 [0.00, 1.00]	0.00 [0.00, 0.00]	0.00254	0.00 [0.00, 0.00]	0.00 [0.00, 0.00]	0.15478
Potentially lethal SA, *n*	1.00 [0.00, 1.00]	0.00 [0.00, 0.00]	0.00033	0.00 [0.00, 0.00]	0.00 [0.00, 1.00]	0.00502
Age at SA1 (%)	26.00 [17.00, 35.00]	20.00 [18.00, 36.00]	0.75222	36.16 (13.87)	33.79 (12.08)	0.67026
FDR mood disorder (%)	99 (55.3)	31 (35.2)	0.0027	76 (73.8)	22 (40.0)	0.0002
FDR bipolar disorder (%)	44 (21.7)	9 (10.1)	0.0214	62 (51.2)	42 (39.3)	0.08039
FDR BD-I, *n*	0.00 [0.00, 0.00]	0.00 [0.00, 0.00]	0.00338	0.00 [0.00, 1.00]	0.00 [0.00, 1.00]	0.05523
FDR BD-II, *n*	0.00 [0.00, 0.00]	0.00 [0.00, 0.00]	0.7157	0.00 [0.00, 0.00]	0.00 [0.00, 0.00]	0.89852
FDR unipolar D, *n*	1.00 [0.00, 1.00]	0.00 [0.00, 1.00]	0.00515	0.00 [0.00, 1.00]	0.00 [0.00, 1.00]	0.5521
FDR SZA, *n*	0.00 [0.00, 0.00]	0.00 [0.00, 0.00]	0.72095	0.00 [0.00, 0.00]	0.00 [0.00, 0.00]	0.7671
FDR SCZ, *n*	0.00 [0.00, 0.00]	0.00 [0.00, 0.00]	0.05084	0.00 [0.00, 0.00]	0.00 [0.00, 0.00]	0.21199
FDR anxiety, *n*	0.00 [0.00, 0.00]	0.00 [0.00, 0.00]	0.00069	0.00 [0.00, 0.00]	0.00 [0.00, 0.00]	0.32271
FDR unaffected, *n*	0.00 [0.00, 1.00]	0.00 [0.00, 0.00]	0.00042	3.50 [0.00, 7.00]	0.00 [0.00, 0.00]	<0.00001
FDR suicide, *n*	0.00 [0.00, 0.00]	0.00 [0.00, 0.00]	0.68103	0.00 [0.00, 0.00]	0.00 [0.00, 0.00]	0.86527
FDR SA, *n*	0.00 [0.00, 0.00]	0.00 [0.00, 0.00]	0.22235	0.00 [0.00, 0.00]	0.00 [0.00, 0.00]	0.07323
SDR suicide, *n*	0.00 [0.00, 0.00]	0.00 [0.00, 0.00]	0.36643	0.00 [0.00, 0.00]	0.00 [0.00, 0.00]	0.66814
SDR SA, *n*	0.00 [0.00, 0.00]	0.00 [0.00, 0.00]	0.26629	0.00 [0.00, 0.00]	0.00 [0.00, 0.00]	0.68636
Mood polarity at suicide attempt (%)			0.33847			1
Major depression	74 (91.4)	3 (75.0)		0 (0.0)	0 (0.0)	
Mania	3 (3.7)	1 (25.0)		3 (16.7)	0 (0.0)	
Minor depression	1 (1.2)	0 (0.0)		0 (0.0)	0 (0.0)	
Mixed	2 (2.5)	0 (0.0)		0 (0.0)	0 (0.0)	
Rapid cycling	1 (1.2)	0 (0.0)		0 (0.0)	0 (0.0)	
LT Hx SI (%)	114 (61.3)	18 (34.0)	0.0007	61 (44.2)	11 (40.7)	0.82612
SI related to mood episode (%)			1			-
No	1 (0.9)	0 (0.0)		0 (0.0)	0 (0.0)	
Sometimes, not always	6 (5.7)	0 (0.0)		0 (0.0)	0 (0.0)	
Yes	99 (93.4)	2 (100.0)		9 (100.0)	8 (100.0)	
Social anxiety disorder (%)	54 (26.6)	0 (0.0)	0.0007	8 (4.5)	26 (35.6)	0.0001
Panic disorder (%)	57 (27.9)	2 (2.1)	0.0001	28 (15.5)	43 (48.9)	0.0001
GAD (%)	84 (41.2)	1 (3.6)	0.0001	13 (7.3)	39 (52.0)	0.0001
OCD (%)	29 (14.1)	2 (2.1)	0.0025	1 (0.6)	8 (9.2)	0.0004
Substance abuse (%)	78 (37.9)	2 (2.0)	0.0001	43 (21.0)	39 (41.5)	0.0005
ADHD (%)	11 (5.5)	0 (0.0)	1	11 (10.6)	45 (60.8)	0.0001
Learning disability (%)	9 (4.5)	0 (0.0)	1	11 (10.6)	38 (51.4)	0.0001
Primary insomnia (%)	35 (17.5)	0 (0.0)	0.38046	7 (6.7)	9 (11.8)	0.28727
Personality disorder (%)	38 (19.1)	0 (0.0)	0.37536	3 (3.4)	23 (31.9)	0.0001
Diabetes mellitus (%)	20 (10.3)	0 (0.0)	0.59934	6 (8.3)	5 (7.5)	1
HTN (%)	22 (11.4)	2 (20.0)	0.61024	17 (23.6)	35 (53.0)	0.0006
Menstrual abnormality (%)	39 (34.2)	3 (60.0)	0.34787	8 (26.7)	2 (4.7)	0.0144
Thyroid disease (%)	55 (29.3)	2 (33.3)	1	18 (32.1)	8 (11.9)	0.0082
Head injury (%)	48 (27.0)	1 (20.0)	1	17 (34.0)	24 (39.3)	0.69833
Migraine (%)	44 (23.5)	2 (33.3)	0.62184	11 (19.3)	9 (13.8)	0.47385
SES (%)			0.0001			0.0001
Disabled	65 (36.3)	1 (3.4)		6 (3.4)	3 (4.0)	
Other	12 (6.7)	8 (27.6)		23 (13.2)	0 (0.0)	
Retired	8 (4.5)	7 (24.1)		25 (14.4)	22 (29.3)	
Social assistance	32 (17.9)	2 (6.9)		4 (2.3)	3 (4.0)	
Unemployment insurance	18 (10.1)	0 (0.0)		7 (4.0)	3 (4.0)	
Unknown	2 (1.1)	1 (3.4)		1 (0.6)	0 (0.0)	
Work full-time	30 (16.8)	10 (34.5)		96 (55.2)	29 (38.7)	
Work part-time	12 (6.7)	0 (0.0)		12 (6.9)	15 (20.0)	
Marital status (%)			0.0001			0.0492
Divorced	47 (23.3)	2 (6.7)		16 (8.1)	9 (11.0)	
Married	84 (41.6)	19 (63.3)		118 (59.6)	51 (62.2)	
Single	67 (33.2)	2 (6.7)		51 (25.8)	11 (13.4)	
Widowed	4 (2.0)	7 (23.3)		13 (6.6)	11 (13.4)	

Categorical data are presented as count (%), whereas normally distributed continuous variables are presented as mean (standard deviation), and non-normal continuous variables are presented as median [interquartile range]. *CU* Cagliari (University), *CB* Cagliari (Centro Bini), *IGSLi* International Group for the Study of Lithium, *MAR* Maritimes, *MTL* Montreal, *ON* Ontario, *POZ* Poznan, *BD* bipolar disorder, *MDD* major depressive disorder, *SZA* schizoaffective disorder, *GFA* global assessment of functioning, *Li* lithium, *SA* suicide attempts, *FDR* first degree relatives, *SDR* second degree relatives, *SCZ* schizophrenia, *SI* suicidal ideation, *Hx* history, *GAD* generalized anxiety disorder, *OCD* obsessive compulsive disorder, *ADHD* attention deficit hyperactivity disorder, *HTN* hypertension, *SES* socioeconomic status.

The LRBest group showed a later age of onset (median 28 y, interquartile range, IQR[21, 36]) compared to NRBest (median 19, IQR;[16, 24] *p* < 0.00001).

All the LRBest subjects for whom clinical course information was available showed a completely episodic course, whereas NRBest courses were mainly chronic fluctuating (43.5%) and episodic with residual symptoms (44.9%; omnibus *p* = 0.0001). Interestingly, *NRPoor* subjects had predominantly completely episodic clinical courses (74.1%), whereas *LRPoor* subjects exhibited predominantly chronic fluctuating (41%) and chronic (25.3%) courses, with only 18.1% being completely episodic (omnibus *p* = 0.0001).

The complete absence of rapid cycling was reported in 98.3% of LRBest, and 47.2% of NRBest (*p* = 0.0001). The majority of NRBest subjects (49.2%) reported spontaneous rapid cycling.

History of lifetime psychosis differed between LRBest and NRBest, with a total of 42.8% of the non-responders reporting episodic and mood congruent psychosis (compared to only 16.7% of responders; *p* = 0.0001). Non-responders also reported incongruent episodic psychosis in 18.6% of cases, with only 37.1% of non-responders reporting an absence of psychosis altogether. In contrast, 83.3% of the best clinical exemplars of lithium response reported a complete absence of lifetime psychosis. There was also a general trend toward lower rates of psychiatric comorbidity in LRBest compared to the NRBest group, whereas LRPoor generally showed more psychiatric comorbidity than NRPoor subjects.

### Step 2: Biological validation through genomic classification

Recall that the genomic data for this element of the analysis are derived from a single site in the ConLiGen data (Dalhousie University, Canada). Supplementary Figs. 3 and 4 demonstrate relative lack of genomic population stratification in this subset, with a comparison to the broader ConLiGen sample.

#### Genomic classification with stratification by clinical exemplar score

Genotyped subjects overlapped with clinical data from the Maritimes (*n* = 129; 40%), Montreal (*n* = 74; 23%), Ontario (*n* = 62; 19%), and IGSLi (*n* = 56; 17%), although in the ConLiGen GWAS^8^, they were all classified as from the Maritimes (Dalhousie University, Canada). It bears repeating that none of the 321 genotyped subjects in the present study came from the relatively genetically distinct Sardinian population whose clinical characteristics were evaluated in Step 1 of our study. Notwithstanding, most clinical differences reflect those reported in Table 1 and thus are reported in Supplementary Table 2.

**Fig. 3:**
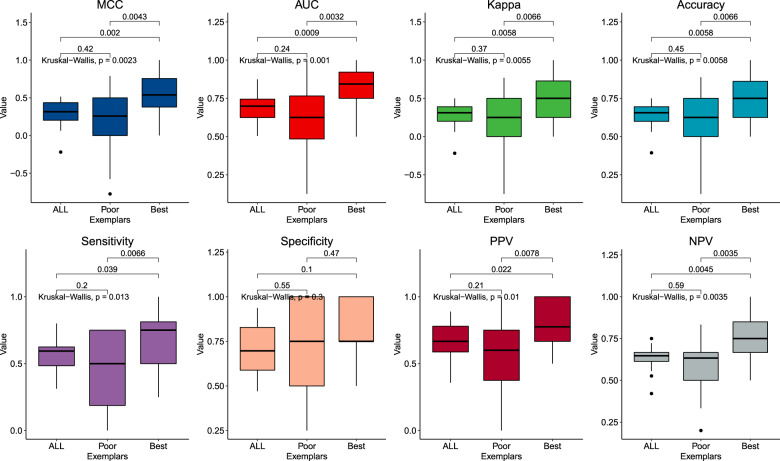
Genomic classification results. Results of classifying lithium response based on the genomic data of all subjects (“ALL”; *n* = 321), the poor clinical exemplars (<25^th^ percentile of clinical exemplar score; *n* = 81), and the best clinical exemplars (>75^th^ percentile of clinical exemplar score; *n* = 79). Boxes are defined by the interquartile range (IQR), with the median shown as the black centered line. Whiskers are 1.5 times the IQR. Each panel shows the results for a different classification performance metric. *MCC* Matthews’ correlation coefficient, *ROC-AUC* area under the receiver operating characteristic curve, *Kappa* Cohen’s kappa, *PPV* positive predictive value, *NPV* negative predictive value.

Genomic classification results are presented in Fig. 3 and in Supplementary Table 3. The median MCC for classification of the best clinical exemplars was 0.58 (IQR [0.41, 0.77]), which was greater than classification analyses with either the poor clinical exemplars (0.29 [0.06, 0.5]; *p* = 0.0043), or the entire dataset (0.32 [0.2, 0.44]; *p* = 0.002). The ROC-AUC for classification of lithium response in the best clinical exemplars was 0.88 [0.83, 0.98], which was greater than that of the model trained only on poor clinical exemplars (0.66 [0.61, 0.80]; *p* = 0.0032) or the whole dataset (0.7 [0.62, 0.75]; *p* = 0.001).

#### Gene enrichment analysis

**Fig. 4:**
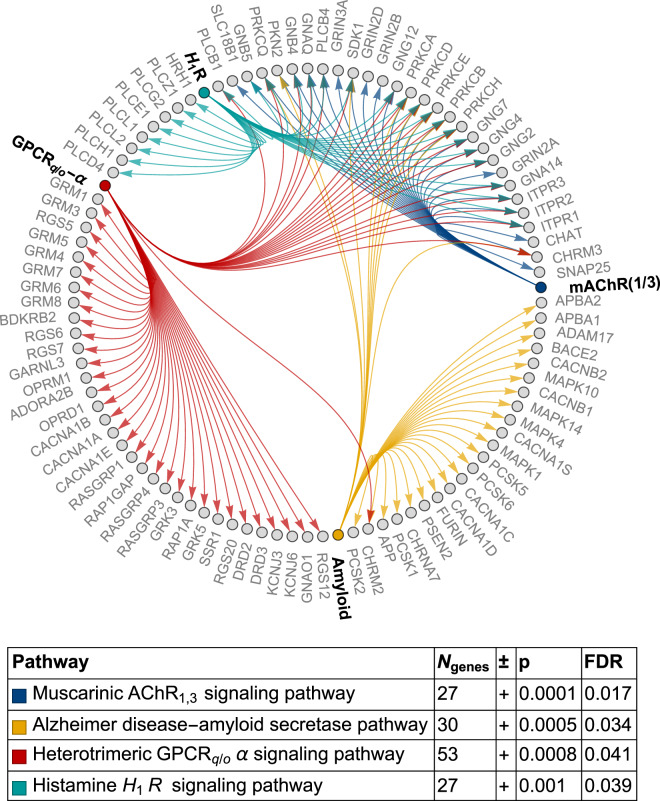
Gene enrichment in the best clinical exemplars. Results of the statistical enrichment test using the logistic regression coefficients from the classifier trained on the best exemplar stratum. Individual genes are shown in gray, with pathway nodes (and edges) colored according to the pathway identity. The terms in bold along the perimeter of the graph (i.e., “Amyloid,” “H_1_R”, “GPCR_q/o_-*α*”, and “mAChR(1/3)”) are pathway names, with edges connected to their constituent genes. *AChR* acetylcholine receptor, *GPCR* G-protein-coupled receptor, *H1R* histamine H1 receptor, *FDR* false discovery rate.

Figure 4 shows pathway analysis results for the best exemplar stratum of the genotyped subjects. Enriched pathways involved (A) muscarinic acetylcholine receptor types 1 and 3 signaling (mAChR1/3; 27 genes, false discovery rate FDR = 0.017), (B) Alzheimer disease-amyloid secretase (30 genes, FDR = 0.034), (C) heterotrimeric G-protein-coupled receptor Gq/Go *α* signaling (GPCRq/o-*α*; 53 genes, FDR = 0.04), and (D) histamine H1R mediated signaling (H1R; 27 genes, FDR = 0.039). Complete gene set analysis results are shown in Supplementary Table 4. Enrichment studies in the gene ontology “cellular component” and “biological function” categories are shown in Supplementary Tables 5 and 6.

## Discussion

This paper has demonstrated that the best clinical exemplars of lithium response and non-response may be more genetically distinct than their less exemplary counterparts, particularly in genes related to GPCRq/o-*α*, mAChR1/3, or H1R signaling, and the Alzheimer’s amyloid–secretase pathway. Clinical exemplars’ clinical profiles were distinct and consistent with past phenotypic research on lithium responders. The genetic separability of clinical exemplars of lithium response and non-response confers some biological validity upon the practice of detailed clinical evaluation, whose predictive utility we have previously demonstrated^5^.

One of our most important findings was characterization of the LRBest group as individuals with generally (A) completely episodic clinical course, (B) few psychiatric comorbidities, (C) later age of onset, (D) absence of rapid cycling, and (E) either absence of psychosis or limitation to mood congruent intra-episodic form. The first two findings are likely the strongest since we observe the opposite pattern among the LRPoor and NRPoor groups. Notwithstanding, all of these elements support past evidence on the clinical phenotype of lithium responsive BD^6,16,17^. For instance, Passmore et al.^18^ found that lithium responders generally had a more episodic course of illness, whereas lamotrigine responders were more likely to have experienced rapid cycling, a higher rate of psychiatric comorbidity, and an earlier age of onset. A later age of onset in lithium responders has been demonstrated in meta-analysis^19,20^. Absence of rapid cycling and psychosis have also been associated with good lithium response^20–23^.

The present study provides strong data-driven support for the idea that a biomarker’s utility is contingent upon the application to patients whose clinical presentations are consistent with the condition being targeted^24^. Furthermore, our study provides biological support for the predictive validity of “classical” clinical pictures of lithium response and non-response^5,6,16,17^. The best clinical exemplars of lithium response and non-response were genetically discriminated with a ROC-AUC of 0.88 (95% CI [0.83,0.98]), whereas we found only a ROC-AUC of 0.66 among poor clinical exemplars (95% CI [0.61,0.80]; *p* = 0.0032). Although the absolute classification performance estimates and their generalizability must be tested on larger, more genetically heterogeneous samples that include distinct subpopulations (for example, Sardinians), our main finding remains important: if there was no biologically mediated information in the exemplary phenotype of lithium response (and non-response), then this difference would not have been observed.

Variants most informative in discrimination of the best exemplars showed enrichment of genes involved in the heterotrimeric GPCRq/o-*α*, mAChR1/3, or H1R signaling, and the Alzheimer’s amyloid–secretase pathway. Lithium response and BD have long been associated with GPCR signaling^25^. In particular, lithium may affect signaling in both the Go–alpha pathway (at least via adenylate cyclase) and the Gq–alpha pathway (via effects on 1,4,5-triphosphate and protein kinase C, PKC)^26–29^. Interestingly, our results imply that differences in GPCR signaling may be segregated according to medication responsiveness. Enrichment in the Alzheimer’s amyloid–secretase pathway is interesting given the growing interest in the effects of lithium on Alzheimer’s pathology. While no significant signal was attributed to the frequently studied gene for *GSK3B* in the pathway analysis, it was involved in the statistically significant enrichment of the glutamatergic synapse cellular component (Supplementary Table 5). It should be noted that the stabilizing effect of lithium likely involves multiple mechanisms, including chronobiological regulations or microRNAs^30–34^. Some of these findings reflect changes in gene expression rather than DNA sequence variation and thus are not directly comparable with our results. In future work, it would be of interest to characterize a more fine-grained “gradient” of genetic differences across the spectrum of clinical exemplar scores.

An important limitation of our study includes the relatively low sample size for the genomic analysis, whose redress is of particular urgency owing to the relatively strong classification performance (AUC 0.88 [0.83, 0.98]). Indeed, small sample sizes in multi-site ML studies may be associated with inflated classification performance^35^, although large counterexamples exist^5,36^. However, our main finding was likely robust to the sample size limitation itself (Supplementary Figs. 6–8). Notwithstanding, we must endeavor to collect detailed clinical information and genotype more patients in our genomic and clinical databases, respectively. As features, our study also used only those SNPs that overlapped across genotyping platforms in the ConLiGen dataset. Unfortunately, however, the number of fully imputed variants was in the order of millions, which would be analytically intractable in the present context. Further discussion of this point is provided in the Supplementary Materials, suffice to say that further methodological work must develop ML methods capable of handling genotypes of 1–10 million SNPs in size.

Our study is also limited by its focus on lithium response, at the exclusion of other mood stabilizers. It is therefore possible, our lithium responders are simply those with a more generally responsive or “less severe” form of BD. The only way to prove specificity would be to obtain data showing a single subject’s non-response to other mood stabilizers and response to lithium, but there is evidence that excellent response to lithium may be exclusive to that medication^37^. Despite their propensity for completely episodic clinical course, lithium responders may also have very severe acute episodes. Finally, individuals with few episodes or infrequent episodes would have lower Alda scores by virtue of the “B”-subscale^38^. It will be of great interest to examine exemplar-based genomic classification of mood stabilizer response more broadly. We are presently collecting clinical and genomic data for patients treated with other mood stabilizers, but these are not yet as abundant as our lithium response data. However, based on our analyses presented here and in our earlier paper^5^, we would (A) recommend lithium as the first-line mood stabilizer for patients with the profile corresponding to exemplary responders (“classical,” Kraepelinean-type BD), (B) not recommend lithium for patients with the characteristics of exemplary non-responders, and (C) consider a time-limited trial of lithium for patients with information insufficient to classify them into either of these two groups.

## Supplementary information

Supplemental materials.
