# Supplementary material for: Exemplar scoring identifies genetically separable phenotypes of lithium responsive bipolar disorder
Source: Transl Psychiatry. 2021 Jan 11;11:36. doi: 10.1038/s41398-020-01148-y (PMC7801503; doi:10.1038/s41398-020-01148-y)
Supplement: Supplementary file 1 — Supplemental materials. [file 41398_2020_1148_MOESM1_ESM.pdf]

Supplemental Materials for *Exemplar Scoring Identifies Genetically Separable Phenotypes of Lithium Responsive Bipolar Disorder*

Abraham Nunes MD PhD MBA, William Stone BSc MSc, Raffaella Ardau MD, Anne Berghöfer MD, Alberto Bocchetta MD, Caterina Chillotti MD, Valeria Deiana MD, Franziska Degenhardt MD, Andreas J. Forstner MD, Julie S. Menzies BN, Eva Grof MD, Tomas Hajek MD PhD, Mirko Manchia MD PhD, Manuel Mattheisen MD, Francis McMahon MD, Bruno Müller-Oerlinghausen MD, Markus M. Nöthen MD, Marco Pinna PsyD, Claudia Pisanu MD, Claire O'Donovan MD, Marcella DC Rietschel MD, Guy Rouleau MD PhD, Thomas Schulze MD, Giovanni Severino MD, Claire M Slaney RN, Alessio Squassina PhD, Aleksandra Suwalska MD, Gustavo Turecki MD PhD, Rudolf Uher MD PhD, Petr Zvolsky MD, Pablo Cervantes MD, Maria del Zompo MD, Paul Grof MD PhD, Janusz Rybakowski MD PhD, Leonardo Tondo MD MSc, Thomas Trappenberg PhD, and Martin Alda MD

Contents

1 Supplementary Methods **2**
1.1 The Clinical Exemplar Score . . . 2
1.2 The Predict Every Subject Out (PESO) Protocol . . . 3
1.3 Gene Set Analysis . . . 3
1.4 Summary of Genomic Preprocessing Methods . . . 5

2 Supplementary Results **5**
2.1 Clinical Data Cohort Descriptions . . . 5
2.2 Evaluation of Population Structure . . . 6
2.3 Classification Performance in the Predict Every Subject Out Analysis . . . 9
2.4 Demographic Comparisons of Best and Poor Exemplars among Genotyped Subjects . . . 10
2.5 Results of Genomic Classification of Lithium Response . . . 14
2.6 Sensitivity Analyses on Genomic Classification . . . 14
2.6.1 Preliminaries . . . 14
2.6.2 Sensitivity to Test-Set Size . . . 15
2.7 Results of Gene Enrichment Analysis . . . 17

3 Supplementary Discussion **25**
3.1 Further Rationale for SNP Set Used in Classification Analyses . . . 25

1 Supplementary Methods

1.1 The Clinical Exemplar Score

Let $(\mathbf{x}_{ij}, y_{ij}) \in \mathcal{X}$ denote phenotypic data from subject $i \in \{1, 2, \dots, n_j\}$, where $\mathbf{x}_{ij}$ is a vector of clinical features, $y_{ij} \in \{0, 1\}$ denotes whether the patient is a lithium responder, and n_j is the number of patients in the sample from site $j \in \{1, 2, \dots, S\}$. A pair $(\mathbf{x}, y)$ can thus be viewed as a set of coordinates on the (observable) phenotypic space $\mathcal{X}$. Data are sampled from S sites, each of which can be considered to sample a subdomain of the phenotypic space $\mathcal{X}^{(j)} \subseteq \mathcal{X}$. These site-wise subdomains are not necessarily disjoint. Indeed, if they were disjoint, the sites' data would share nothing in common.

Now let $\mathcal{M}_j$ denote a classifier learned on training data from site j. Given a new set of clinical features, $\mathbf{x}'$, the classifier predicts the probability that the corresponding patient is a lithium responder: that is, $\hat{p}'_j = \mathcal{M}_j(\mathbf{x}')$. We denote the accuracy score of this prediction as

$$\tilde{f}_j(\mathbf{x}', y') = 1 - |y' - \mathcal{M}_j(\mathbf{x}')| \,. \tag{1}$$

The representational Rényi heterogeneity measurement approach (1) consists of measuring heterogeneity on a latent or transformed space onto which observable data are mapped. To apply this in the present case, where we have defined our observable space, $\mathcal{X}$, we must now devise an appropriate transformed space upon which the Rényi heterogeneity will be both meaningful and tractable. The heterogeneity deemed relevant in the present case arises in terms of differences in classification models across sites. Most starkly, we noted that the informative features for lithium response prediction varied between the best performing sites. In other words, depending on which site's data are used for training, one might learn quite different (and perhaps even contradictory) relationships between clinical features and lithium responsiveness. In the limit where data from each site encodes completely different relationships between clinical features and lithium response, then each classifier $\mathcal{M}_j$ will behave distinctly (they will tend to disagree). In terms of numbers equivalent, we would say that in such a case there is an effective number of S distinct classifiers. Conversely, if the phenotypic domains of all sites overlap completely, then all classifiers $\mathcal{M}_j$ will tend to make similar predictions, which would correspond to an effective number of one classifier.

Let the accuracy of classifier $\mathcal{M}_j$ in predicting the relationship $\mathbf{x} \to y$ be a measure of that model's informativeness at point $(\mathbf{x}, y)$. We can thus define $\mathcal{T}$ as a categorical space representing an index on "the most informative classifier." We illustrate the mapping $f : \mathcal{X} \to \mathcal{T}$ in Figure 1. A probability distribution over $\mathcal{T}$ can be computed using a normalization of Equation 1:

$$f(\mathbf{x}, y) = \left\{ \frac{1 - |y - M_j(\mathbf{x})|}{\sum_{k=1}^{S} (1 - |y - M_k(\mathbf{x})|)} \right\}_{j=1}^{S} . \tag{2}$$

The quantity $f_j(\mathbf{x}, y)$ can be taken to represent the probability that a classifier trained on data from site j is the most informative about the $\mathbf{x} \to y$ mapping in that particular region of $\mathcal{X}$. With this, we can compute the representational

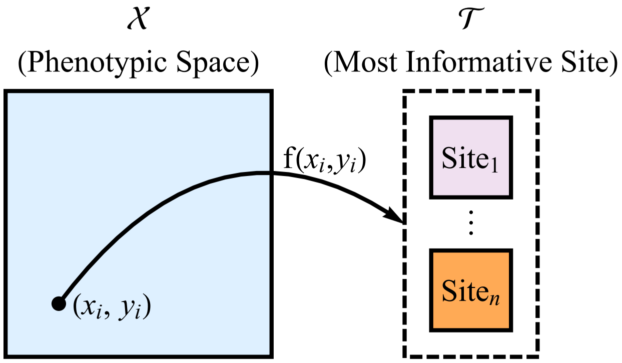

Supplementary Figure 1: Representation of the mapping from phenotypic space $\mathcal{X}$ onto the representation of "most informative site-level model" ($\mathcal{T}$). The transformation function is the normalized accuracy score for a classification model trained on each site's data individually (Equation 2).

Rényi heterogeneity at $(\mathbf{x}, y)$ as follows:

$$\Pi_q(\mathbf{x}, y) = \left(\sum_{j=1}^{S} f_j^q(\mathbf{x}, y) \right)^{\frac{1}{1-q}}. \tag{3}$$

If the models $\mathcal{M}_{j=1,2,\dots,S}$ differ only in their training data (i.e. they have the same architecture, optimization routine, and hyperparameters) then the units of Equation 3 are "the effective number of informative sites."

Recall that we defined a "clinical exemplar" as a subject whose phenotype $(\mathbf{x}, y)$ is reliably predicted accurately across all sites. In other words, regardless of the differences between sites' data, all sites would agree in their predictions of the exemplars' phenotypes. More formally, clinical exemplars must have high values of $\Pi_q(\mathbf{x}, y)$ (all sites are similarly informative). However, to identify more specifically the exemplars of lithium response and non-response, we cannot solely rely on $\Pi_q(\mathbf{x}, y)$, since that value may be high, despite sites' prediction accuracies being low.

Let $t_* = \max_j \tilde{f}_j(\mathbf{x}, y)$ denote the maximal accuracy score obtained in classification at $(\mathbf{x}, y)$. We take this value to represent the degree to which a subject with that phenotype can be clearly associated with one class or another. An interesting case occurs where both t_* and $\Pi_q(\mathbf{x}, y)$ are high, suggesting the point $(\mathbf{x}, y)$ is an exemplar of the regions of $\mathcal{X}$ that are reliably well classified across sites. Conversely, if $t_* \approx 0.5$ and $\Pi_q(\mathbf{x}, y)$ is high, then that point is exemplary of a region of $\mathcal{X}$ of which all sites are uncertain. When t_* is low and $\Pi_q(\mathbf{x}, y)$ is high, then $(\mathbf{x}, y)$ is exemplary of a region of $\mathcal{X}$ that reliably misleads all sites' classifiers.

In the present study, we are concerned with identifying only those subjects with high values of both t_* and $\Pi_q(\mathbf{x}, y)$, since they exemplify the most canonical "phenotypes" of lithium response and non-response, respectively. We accomplish this by combining t_* and $\Pi_q(\mathbf{x}, y)$ into a single index we call the *exemplar score*. The exemplar score at coordinate $(\mathbf{x}, y)$ of the phenotypic space is defined as

$$\phi = \sqrt{\frac{\tilde{\Pi}_q^2(\mathbf{x}, y) + (t^*)^2}{2}}, \tag{4}$$

where $\tilde{\Pi}_q(\mathbf{x}, y)$ is a standardization of the Rényi heterogeneity to the [0,1] interval (the same scale as t^*):

$$\tilde{\Pi}_q(\mathbf{x}, y) = \frac{\Pi_q(\mathbf{x}, y) - 1}{S - 1} \tag{5}$$

In the present study, we define the "best exemplars" as subjects whose exemplar scores (within their lithium response classes) were in the top 25%. Poor exemplars were those subjects whose phenotypes were in the lower quartile of exemplar scores within their response classes.

1.2 The Predict Every Subject Out (PESO) Protocol

The predict every subject out protocol (PESO; Figure 2) is a method by which we can compute exemplar scores for each subject in the dataset while (A) ensuring that subject is not included in the training data and (B) having each model train on only that site's data. All classifiers in our data were random forests, (RFC) (2) under the same specifications as in Nunes et al. (3) (100 estimators; SciKit Learn implementation; (4)). Similar to that study, missing data were marginalized by sampling from uninformative priors on respective variables' domains (3).

For each site in the clinical predictors dataset, the PESO analysis protocol begins with a Leave-One-Out cross-validation run to obtain out-of-sample predictions for each of that site's constituent subjects. We then train an RFC on that site's data and predict lithium response in all other sites' subjects. Each subject is thus mapped onto our categorical space $\mathcal{T}$, upon which we can measure their exemplar scores.

1.3 Gene Set Analysis

At each fold of cross-validation, the logistic regression coefficients were saved. The SNPs whose logistic regression coefficients were of the same sign (i.e. positive or negative, such that we focus only on SNPs with consistent associations) across all folds were ranked in terms of their absolute median coefficient values and linked to gene identifiers using the NCBI gene database. Each gene was assigned the maximal absolute value of the logistic regression coefficients for all SNPs tagged by that gene; the remainder (duplicates) were deleted, such that each included gene had only one numerical value associated with it. We then applied the statistical enrichment test in the PANTHER classification system v. 14.1

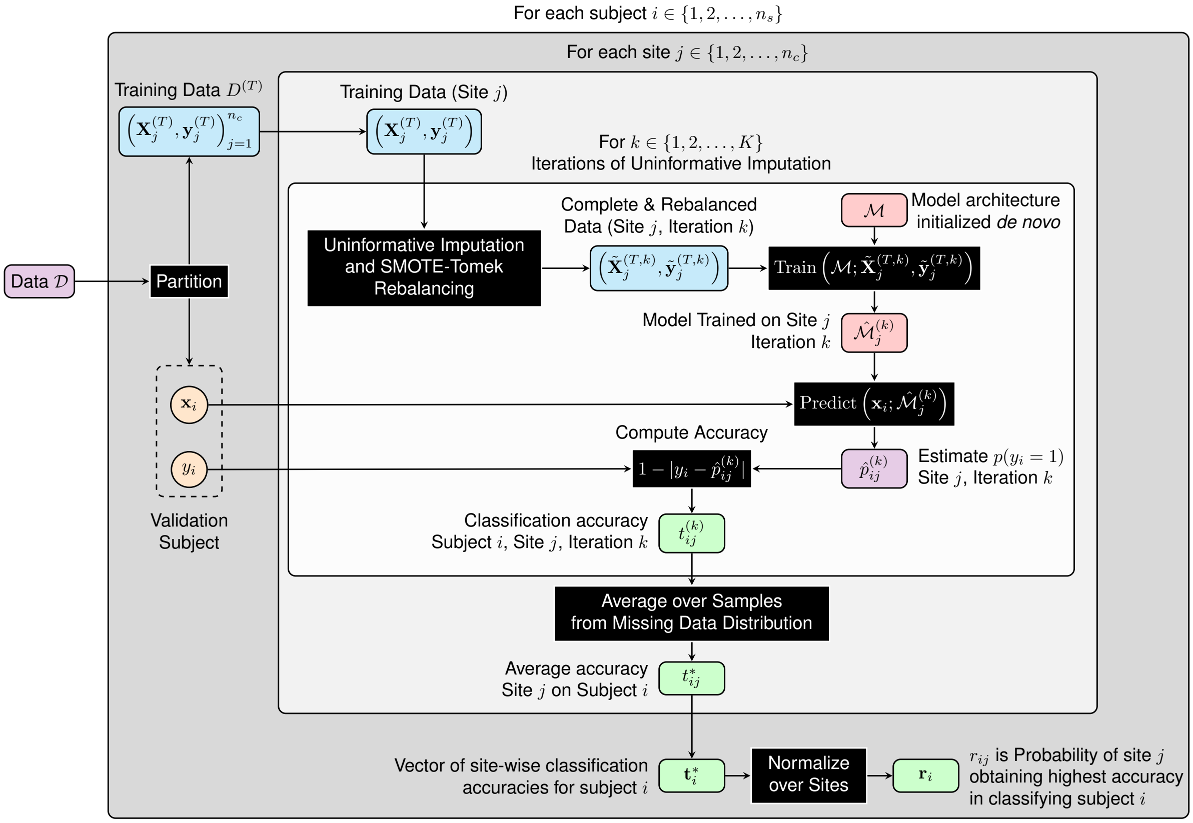

Supplementary Figure 2: Illustration of the algorithm for the predict every subject out protocol.

Supplementary Table 1: Description of constituent datasets. *Abbreviations*: number of patients (N), lithium responders (LR+), Cagliari (Centro Bini; CB), Cagliari (University; CU), International Group for the Study of Lithium (IGSLi), Maritimes (MAR), Ontario (ON), Poznan (POZ).

Sample	N (LR+)	Description
**CB**	324 (21%)	Patients followed at the Mood Disorder Lucio Bini Center in Cagliari, Italy. Clinical data collection and response assessment was done by two psychiatrists.
**CU**	206 (29%)	Patients in the long term treatment program at the Lithium Clinic of the Unit of the Clinical Pharmacology Center, University Hospital of Cagliari, Italy. Clinical data collection and response assessment was done by three psychiatrists and three clinical psychopharmacologists.
**IGSLi**	70 (100%)	Patients recruited for a genetic study of lithium responsive bipolar disorder. (7) By design of that study, all patients were lithium responders. Clinical data collection and response assessment was done by three psychiatrists.
**MAR**	343 (20%)	Patients followed by the Mood Disorders program at the Nova Scotia Health Authority and the Maritime Bipolar Registry. Clinical data collection and response assessment was done by two psychiatrists and two research nurses working in pairs.
**MTL**	95 (16%)	Patients followed by the Mood Disorders Program at the McGill University Health Centre. Clinical data collection and response assessment was done by one psychiatrist.
**ON**	117 (84%)	Patients from our earlier studies of lithium responsive bipolar disorder, (7, 8) which, like the IGSLi sample, explains the greater proportion of responders. Clinical data collection and response assessment was done by three psychiatrists (including MA, who is now in the Maritimes).
**POZ**	111 (53%)	Patients followed longitudinally by the Psychiatry Department at the University of Poznan, Poland. Clinical data collection and response assessment was done by two psychiatrists.

(5). We repeated the statistical enrichment test for the following annotation sets: PANTHER pathways, GO molecular function (complete), GO biological processes (complete), GO cellular components (complete). To further evaluate the degree to which the enrichment analyses speak specifically to findings among the best exemplars, we repeated the same procedures outlined here using the logistic regression coefficients for the poor exemplars.

1.4 Summary of Genomic Preprocessing Methods

Our raw dataset consisted of the genotypes resulting from the preprocessing and imputation steps taken by Hou et al. (6). We summarize their quality control and imputation steps here. However, note that the sample used for the present study includes only SNPs that were directly genotyped across all sites. Our subject sample is restricted only to those from the Dalhousie University sample of ConLiGen, since these were the only such subjects for whom clinical variables were also available.

Hou et al. (6) provided the following quality control parameters for retaining SNPs and subjects. Subject-wise SNP-missingness rate less than 0.03. The autosomal heterozygosity rate was within a mean of +/- 3 standard deviations. Minor allele frequency must have been greater than 0.01. Missingness (SNP-wise) must have been less than a rate of 0.05. The SNP Hardy-Weinberg equilibrium p-values were greater than 10^{-4} in all samples. Hou et al. (6) detected no discrepancies between reported and genotypic sex.

2 Supplementary Results

2.1 Clinical Data Cohort Descriptions

Descriptions of the dataset of clinical variables is provided in Table 1.

2.2 Evaluation of Population Structure

To evaluate for the presence of population stratification in our genomic sample, we plot the first several principal components of the subjects' genotypes in Figure 3. For comparison, Figure 4 demonstrates the first several principal components from 14 sites of the full Consortium on Lithium Genetics (ConLiGen) genomic sample.

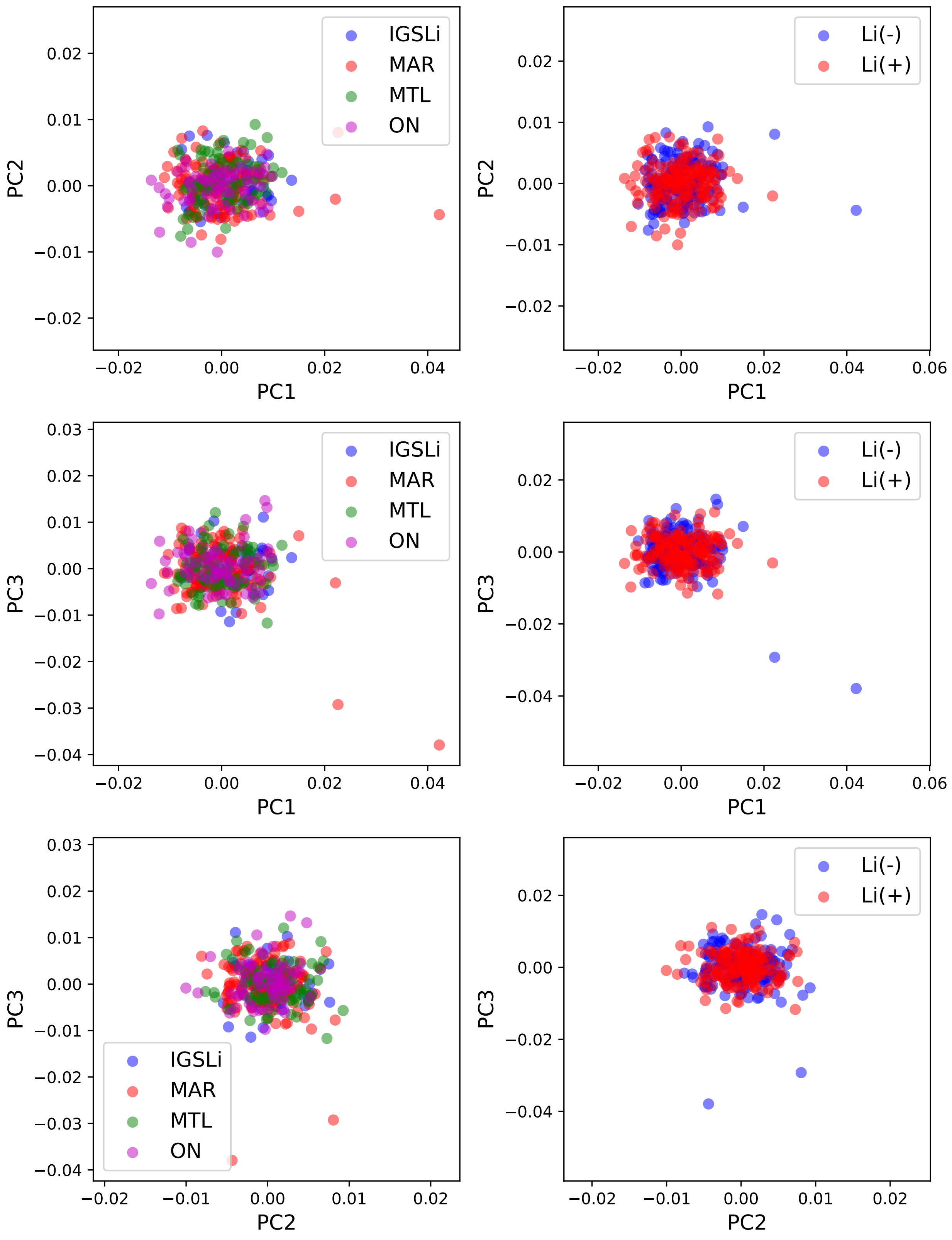

Supplementary Figure 3: Principal components analysis of the genomic dataset from Halifax (as coded in the ConLiGen studies (6)). The left column is coloured by the site of origin, whereas the right column of plots is coloured by lithium responsiveness. *Abbreviations*: International Group for the Study of Lithium (IGSLi), Maritimes (MAR), Montreal (MTL), Ontario (ON; also known as Ottawa/Hamilton).

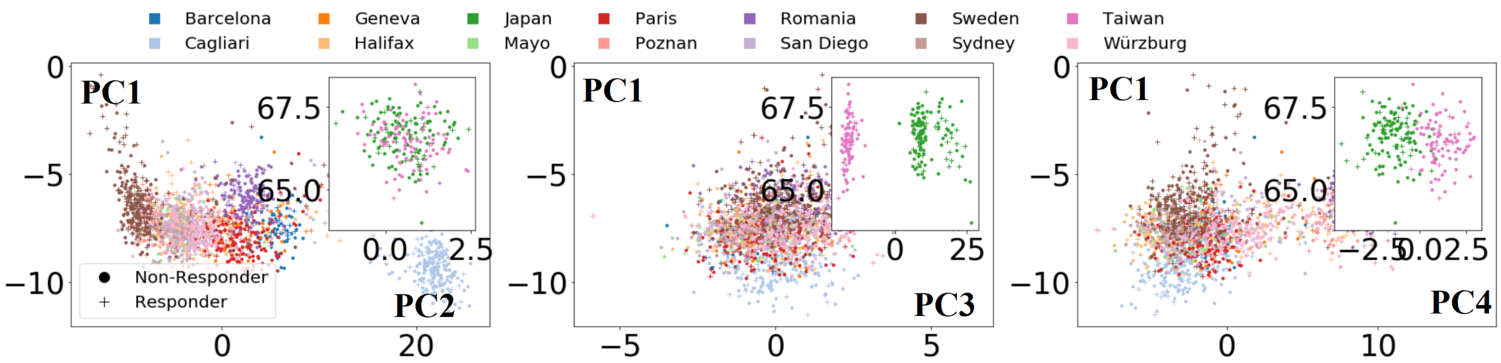

Supplementary Figure 4: Principal components analysis of the genomic dataset from the Consortium on Lithium Genetics sample (6).

2.3 Classification Performance in the Predict Every Subject Out Analysis

Figure 5 plots the site-level models' accuracy distributions. This plot shows that the site-level accuracies were highly variable in shape and modality. This provides further confirmation that classification behaviour between site-level models was heterogeneous. Several of the distributions shown in Figure 5 can be easily appreciated as corresponding to the Brier scores reported in evaluation of model calibraion by Nunes et al. (3). For example, the Brier score for the Maritimes clinical dataset was 0.15 (95% CI 0.13-0.16), whereas for the Poznan site it was 0.24 (0.23-0.24) in the original study. Figure 5 indeed shows that the probabilistic predictions made by the Maritimes site are more widely distributed than those of Poznan, as one would expect with a better calibrated model. One can also appreciate the limitations inherent to IGSLi's inclusion of only lithium responders (which in Nunes et al. (3) prevented reporting of a site-level analysis for this sample). That is, since IGSLi includes only lithium responders, it achieves perfect accuracy only for the lithium responders, with completely erroneous responses for the non-responders.

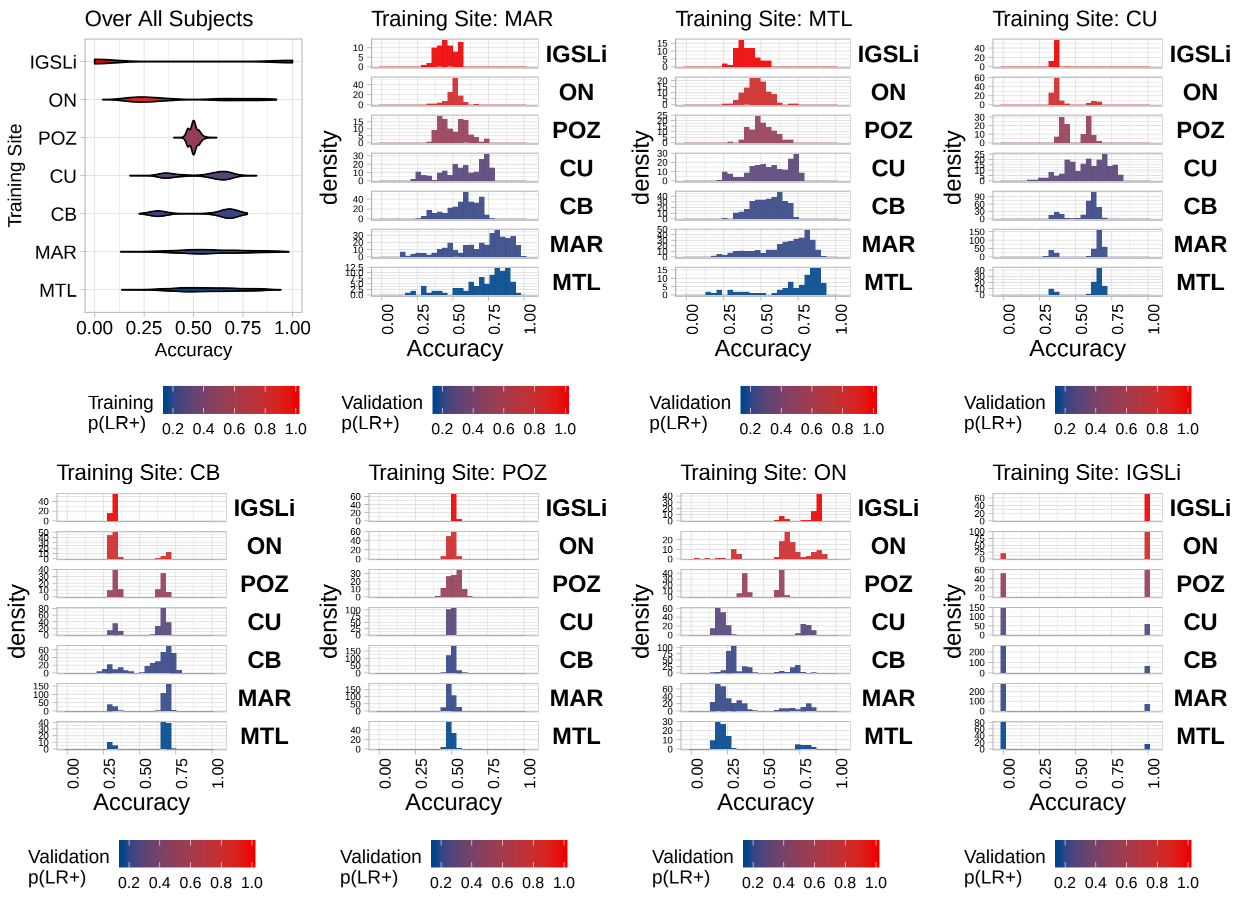

Supplementary Figure 5: Accuracy distributions for models evaluated under the predict every subject out (PESO) regime. The violin plot at the upper leftmost corner shows the accuracy distributions for each site model evaluated over all subjects in the dataset, with the densities colored according to the proportion of lithium responders in the training site's data. The remaining subplots show accuracy histograms for training site models (specified in the titles) stratified across out-of-sample sites. For the site-wise histograms, color indicates the responder/non-responder balance in the respective validation site. Abbreviations: Lithium responder (LR+), Cagliari (Centro Bini; CB), Cagliari (University; CU), International Group for the Study of Lithium (IGSLi), Maritimes (MAR), Ontario (ON), Poznan (POZ).

2.4 Demographic Comparisons of Best and Poor Exemplars among Genotyped Subjects

Clinical demographic comparisons between the best exemplars, poor exemplars, and the aggregated sample of genotyped patients is presented in Table 2, with stratification by lithium response. The results of gene enrichment analysis are presented in Table 4, with specific genes enriched in the best exemplar group (related to glutamate receptors and signalling processes) shown in Tables 5 and 6.

Supplementary Table 2: Demographic comparisons for subjects whose genomic data (from the Consortium for Lithium Genetics; ConLiGen) overlapped with our clinical dataset. Comparisons were done in between lithium responders (LR(+)) and non-responders (LR(-)) for the total group ("ALL), the best exemplars ("Best; exemplar score $\geq$ 75th percentile), and the poorest exemplars ("Poor; exemplar score $\leq$ 25th percentile). Abbreviations: Calgiari (University; CU), Cagliari (Centro Bini; CB), International Group for the Study of Lithium (IGSLi), Maritimes (MAR), Montreal (MTL), Ontario (ON), Poznan (POZ), bipolar disorder (BD), major depressive disorder (MDD), lifetime (LT), antidepressants (AD), schizoaffective disorder (SZA), global assessment of functioning (GAF), lithium (Li), suicide attempts (SA), first degree relatives (FDR), second degree relatives (SDR), schizophrenia (SCZ), suicidal ideation (SI), history (Hx), generalized anxiety disorder (GAD), obsessive compulsive disorder (OCD), substance use disorder (SUD), attention deficit hyperactivity disorder (ADHD), learning disability (LD), personality disorder (PD), hypertension (HTN), traumatic brain injury (TBI), socioeconomic status (SES).

	ALL			Poor			Best		
	**LR(-)**	**LR(+)**	**p**	**LR(-)**	**LR(+)**	**p**	**LR(-)**	**LR(+)**	**p**
n	162	159		41	40		40	39	
Centre (%)			<0.001			<0.001			<0.001
IGSLi	0 (0.0)	56 (35.2)		0 (0.0)	8 (20.0)		0 (0.0)	33 (84.6)	
Maritimes	92 (56.8)	37 (23.3)		22 (53.7)	10 (25.0)		23 (57.5)	0 (0.0)	
Montreal	62 (38.3)	12 (7.5)		14 (34.1)	3 (7.5)		17 (42.5)	0 (0.0)	
Ontario	8 (4.9)	54 (34.0)		5 (12.2)	19 (47.5)		0 (0.0)	6 (15.4)	
GWAS Wave 2 (%)	93 (57.4)	20 (12.6)	<0.001	22 (53.7)	5 (12.5)	<0.001	27 (67.5)	0 (0.0)	<0.001
Male (%)	66 (40.7)	70 (44.0)	0.629	19 (46.3)	18 (45.0)	1	16 (40.0)	16 (41.0)	1
Age (y)	48.53 [21.59, 82.51]	50.94 [21.66, 80.16]	0.009	49.43 (14.47)	52.63 (13.83)	0.312	42.50 (12.81)	59.10 (11.07)	<0.001
Diagnosis (%)			0.154			0.015			0.11
BD I	108 (66.7)	112 (70.4)		34 (82.9)	20 (50.0)		24 (60.0)	26 (66.7)	
BD II	52 (32.1)	41 (25.8)		7 (17.1)	18 (45.0)		16 (40.0)	9 (23.1)	
MDD Recurrent	0 (0.0)	4 (2.5)		0 (0.0)	1 (2.5)		0 (0.0)	3 (7.7)	
MDD Single	0 (0.0)	1 (0.6)		0 (0.0)	1 (2.5)		0 (0.0)	0 (0.0)	
SZA	2 (1.2)	1 (0.6)		0 (0.0)	0 (0.0)		0 (0.0)	1 (2.6)	
Age of Onset (y)	22. [7., 64.]	26. [13., 63.]	<0.001	25. [12., 54.]	25. [14., 48.]	0.429	17. [7., 30.]	28. [17., 63.]	<0.001
Onset Dep. (y)	24. [12., 67.]	29. [14., 63.]	<0.001	30.29 (10.08)	27.62 (8.60)	0.243	20. [12., 35.]	32. [19., 63.]	<0.001
Onset M (y)	29. [15., 59.]	30. [13., 66.]	0.292	30. [17., 56.]	32. [18., 66.]	0.193	27.50 [15., 47.]	32. [17., 52.]	0.039
Onset HypoM (y)	30. [0., 67.]	36.50 [16., 63.]	0.254	31. (13.39)	35.74 (11.36)	0.253	24. [12., 62.]	38. [20., 63.]	0.054
Polarity 1st Ep. (%)			0.006			0.118			0.073
Biphasic (D-M)	2 (1.2)	9 (5.9)		1 (2.6)	3 (8.3)		1 (2.5)	3 (7.9)	
Biphasic (M-D)	10 (6.2)	8 (5.3)		3 (7.7)	2 (5.6)		3 (7.5)	1 (2.6)	
Hypomania	18 (11.2)	13 (8.6)		6 (15.4)	2 (5.6)		5 (12.5)	2 (5.3)	
Major dep.	99 (61.9)	68 (44.7)		14 (35.9)	21 (58.3)		28 (70.0)	21 (55.3)	
Mania	20 (12.5)	36 (23.7)		12 (30.8)	4 (11.1)		1 (2.5)	7 (18.4)	
Minor dep.	7 (4.4)	16 (10.5)		2 (5.1)	4 (11.1)		1 (2.5)	4 (10.5)	
Mixed	2 (1.2)	1 (0.7)		1 (2.6)	0 (0.0)		1 (2.5)	0 (0.0)	
Periodic rapid cyc.	2 (1.2)	1 (0.7)		0 (0.0)	0 (0.0)		0 (0.0)	0 (0.0)	
Clinical Course (%)			<0.001			0.01			NaN
Chronic	7 (4.5)	0 (0.0)					1 (2.5)	0 (0.0)	
Chronic fluctuating	49 (31.6)	6 (12.2)		3 (8.1)	4 (30.8)		19 (47.5)	0 (0.0)	
Completely episodic	41 (26.5)	35 (71.4)		29 (78.4)	4 (30.8)		0 (0.0)	0 (0.0)	
Episodic + Residual	56 (36.1)	7 (14.3)		4 (10.8)	5 (38.5)		20 (50.0)	0 (0.0)	
Single episode	2 (1.3)	1 (2.0)		1 (2.7)	0 (0.0)		0 (0.0)	0 (0.0)	
N LT Manias	2. [0., 99.]	2. [0., 34.]	0.052	2. [0., 11.]	1. [0., 25.]	0.041	2. [0., 99.]	1. [0., 8.]	0.103
N LT Dep.	4. [0., 99.]	3. [0., 35.]	0.001	3. [0., 27.]	3. [0., 25.]	0.277	7. [0., 99.]	3. [0., 15.]	<0.001
N LT Mixed	0. [0., 99.]	0. [0., 3.]	<0.001	0. [0., 1.]	0. [0., 2.]	0.403	0. [0., 99.]	0. [0., 0.]	<0.001
N LT Multiphasic	0. [0., 99.]	0. [0., 13.]	0.454	0. [0., 1.]	0. [0., 9.]	0.109	1. [0., 99.]	0. [0., 13.]	0.203
Total LT Episodes	8. [1., 99.]	6. [0., 99.]	0.007	6. [1., 33.]	6. [0., 50.]	0.899	11.50 [3., 99.]	6.50 [2., 27.]	0.002
Rapid Cycling (%)			<0.001			0.798			<0.001
Never	92 (60.1)	104 (94.5)		0 (0.0)	0 (0.0)		12 (30.0)	25 (100.0)	
Only on AD	6 (3.9)	0 (0.0)		0 (0.0)	0 (0.0)		2 (5.0)	0 (0.0)	

Continued on next page...

Supplementary Table 2: Continued

	ALL			Poor			Best		
	LR(-)	LR(+)	p	LR(-)	LR(+)	p	LR(-)	LR(+)	p
Spontaneous	55 (35.9)	6 (5.5)		1 (2.8)	2 (7.4)		26 (65.0)	0 (0.0)	
Rapid mood switch	46 (54.1)	4 (21.1)	0.019	6 (30.0)	1 (20.0)	1	14 (63.6)	0 (0.0)	-
LT Psychosis (%)			0.888			0.469			0.659
Episodic congruent	57 (37.7)	27 (42.9)		14 (37.8)	9 (50.0)		18 (45.0)	0 (0.0)	
Episodic incong.	21 (13.9)	7 (11.1)		6 (16.2)	1 (5.6)		6 (15.0)	0 (0.0)	
Never	70 (46.4)	28 (44.4)		17 (45.9)	8 (44.4)		15 (37.5)	1 (100.0)	
Out of episodes	3 (2.0)	1 (1.6)					1 (2.5)	0 (0.0)	
GAF last Ax	70. [35., 95.]	90. [0., 100.]	<0.001	80. [50., 95.]	90. [40., 95.]	0.006	70. [40., 90.]	90. [90., 95.]	<0.001
Total ALDA Score	2. [0., 6.]	8. [7., 10.]	<0.001	4. [0., 6.]	8. [7., 10.]	<0.001	2. [0., 6.]	8. [8., 10.]	<0.001
N Episodes on Li	3. [0., 99.]	0. [0., 5.]	<0.001	2.50 [0., 99.]	0. [0., 2.]	0.002	3.50 [1., 99.]	-	-
N Episodes Pre-Li	4. [1., 99.]	4.50 [2., 99.]	0.373	3. [1., 99.]	5. [2., 99.]	0.078	8.50 [1., 99.]	-	-
N SA	0. [0., 6.]	0. [0., 3.]	0.119	0. [0., 2.]	0. [0., 2.]	0.235	1. [0., 6.]	0. [0., 3.]	0.022
N serious SA	1. [0., 6.]	0.50 [0., 2.]	0.044	1. (0.82)	1. (1.00)	1	1. [0., 3.]	0. [0., 0.]	0.177
Age First SA (y)	27.50 [12., 64.]	30.50 [16., 55.]	0.308	39.75 (12.69)	34. (10.39)	0.552	24.30 (9.60)	-	-
N FDR Mood d/o	75 (57.7)	61 (41.2)	0.009	18 (60.0)	11 (30.6)	0.031	24 (70.6)	16 (42.1)	0.028
FDR BD (%)	56 (34.6)	41 (25.9)	0.12	20 (48.8)	14 (35.9)	0.348	10 (25.0)	0 (0.0)	0.003
N FDR BD-I	0. [0., 4.]	0. [0., 5.]	0.13	0. [0., 4.]	0. [0., 5.]	0.3	0. [0., 2.]	0. [0., 0.]	0.001
N FDR MDD	1. [0., 7.]	0. [0., 5.]	0.01	0. [0., 7.]	0. [0., 3.]	0.158	1. [0., 3.]	0. [0., 5.]	0.019
N FDR SZA	0. [0., 1.]	0. [0., 1.]	0.678	0. [0., 1.]	0. [0., 0.]	0.165	0. [0., 1.]	0. [0., 1.]	0.986
N FDR SCZ	0. [0., 2.]	0. [0., 1.]	0.01	0. [0., 2.]	0. [0., 1.]	0.216	0. [0., 2.]	0. [0., 0.]	0.127
N FDR Ans d/o	0. [0., 3.]	0. [0., 3.]	0.044	0. [0., 2.]	0. [0., 1.]	0.129	0. [0., 2.]	0. [0., 1.]	0.006
N FDR Unaff.	0. [0., 5.]	0. [0., 2.]	<0.001	0. [0., 3.]	0. [0., 1.]	0.014	0. [0., 4.]	0. [0., 1.]	<0.001
N FDR Suicide	0. [0., 2.]	0. [0., 2.]	0.801	0. [0., 1.]	0. [0., 2.]	0.384	0. [0., 1.]	0. [0., 0.]	0.779
N FDR SA	0. [0., 2.]	0. [0., 2.]	0.193	0. [0., 2.]	0. [0., 2.]	0.183	0. [0., 2.]	0. [0., 0.]	0.743
N SDR Suicide	0. [0., 1.]	0. [0., 2.]	0.738	0. [0., 1.]	0. [0., 0.]	0.232	0. [0., 1.]	0. [0., 0.]	0.819
N SDR SA	0. [0., 1.]	0. [0., 1.]	0.387	0. [0., 1.]	0. [0., 0.]	0.499	0. [0., 1.]	0. [0., 0.]	0.819
LT Hx SI	73 (54.5)	27 (38.0)	0.036	8 (29.6)	6 (37.5)	0.845	32 (82.1)	7 (43.8)	0.012
SI episode related (%)			0.284			-			1
No	1 (1.4)	1 (6.7)		0 (0.0)	0 (0.0)		0 (0.0)	0 (0.0)	
Sometimes	5 (7.1)	0 (0.0)		0 (0.0)	0 (0.0)		0 (0.0)	0 (0.0)	
Yes	64 (91.4)	14 (93.3)		8 (100.0)	5 (100.0)		26 (89.7)	1 (100.0)	
Social Anx. d/o (%)	28 (18.3)	6 (12.8)	0.508	2 (5.7)	3 (25.0)	0.184	8 (20.0)	0 (0.0)	1
Panic d/o (%)	32 (20.6)	5 (4.3)	<0.001	1 (2.8)	2 (7.7)	0.772	12 (30.0)	0 (0.0)	0.001
GAD (%)	37 (24.2)	3 (6.4)	0.014	4 (11.4)	1 (8.3)	1	14 (35.0)	0 (0.0)	1
OCD (%)	13 (8.4)	1 (0.8)	0.011	1 (2.8)	1 (3.8)	1	6 (15.0)	0 (0.0)	0.039
SUD (%)	43 (27.7)	20 (16.4)	0.036	6 (16.7)	7 (25.0)	0.611	14 (35.0)	1 (2.6)	0.001
ADHD (%)	12 (7.8)	1 (2.2)	0.31	5 (13.9)	1 (7.7)	0.928	3 (7.5)	0 (0.0)	-
LD (%)	7 (4.6)	1 (2.2)	0.765	2 (5.7)	0 (0.0)	0.946	2 (5.0)	0 (0.0)	-
Insom (%)	18 (11.7)	3 (6.7)	0.491	2 (5.6)	0 (0.0)	0.96	6 (15.0)	0 (0.0)	-
PD (%)	34 (22.2)	4 (8.7)	0.067	2 (5.6)	0 (0.0)	0.96	13 (33.3)	0 (0.0)	-
Diabetes (%)	22 (14.7)	4 (9.3)	0.512	4 (11.4)	0 (0.0)	0.575	6 (15.4)	0 (0.0)	-
HTN (%)	25 (16.9)	6 (14.3)	0.867	5 (14.7)	2 (20.0)	1	5 (13.2)	0 (0.0)	-
Menstrual abn (%)	22 (28.9)	8 (42.1)	0.408	1 (7.1)	3 (75.0)	0.028	4 (17.4)	0 (0.0)	-
Thyroid d/o (%)	51 (34.5)	16 (37.2)	0.88	13 (37.1)	4 (36.4)	1	13 (33.3)	0 (0.0)	-
TBI (%)	27 (20.9)	7 (20.0)	1	6 (21.4)	4 (44.4)	0.357	8 (22.2)	0 (0.0)	-
Migraine (%)	41 (29.1)	4 (9.1)	0.013	8 (25.0)	1 (8.3)	0.423	8 (21.1)	0 (0.0)	-
SES (%)			0.181			0.113			-
Work full-time	27 (19.3)	11 (23.4)		14 (42.4)	3 (23.1)		5 (12.8)	0 (0.0)	
Work part-time	12 (8.6)	7 (14.9)		1 (3.0)	2 (15.4)		4 (10.3)	0 (0.0)	
Unemployment ins	20 (14.3)	4 (8.5)		6 (18.2)	1 (7.7)		4 (10.3)	0 (0.0)	
Social assist.	19 (13.6)	6 (12.8)		0 (0.0)	2 (15.4)		9 (23.1)	0 (0.0)	
Disabled	34 (24.3)	7 (14.9)		5 (15.2)	2 (15.4)		9 (23.1)	0 (0.0)	
Other	3 (2.1)	4 (8.5)		0 (0.0)	0 (0.0)		1 (2.6)	0 (0.0)	

Continued on next page...

Supplementary Table 2: Continued

	ALL			Poor			Best		
	**LR(-)**	**LR(+)**	**p**	**LR(-)**	**LR(+)**	**p**	**LR(-)**	**LR(+)**	**p**
Retired	19 (13.6)	8 (17.0)		7 (21.2)	3 (23.1)		2 (5.1)	0 (0.0)	
Student	6 (4.3)	0 (0.0)		0 (0.0)	0 (0.0)		5 (12.8)	0 (0.0)	
Marital status (%)			0.547			0.444			-
Single	34 (23.3)	12 (23.1)		3 (9.4)	4 (25.0)		17 (42.5)	0 (0.0)	
Married	76 (52.1)	32 (61.5)		19 (59.4)	9 (56.2)		13 (32.5)	0 (0.0)	
Divorced	32 (21.9)	7 (13.5)		9 (28.1)	3 (18.8)		9 (22.5)	0 (0.0)	
Widowed	4 (2.7)	1 (1.9)		1 (3.1)	0 (0.0)		1 (2.5)	0 (0.0)	

2.5 Results of Genomic Classification of Lithium Response

Supplementary Table 3: Results of classifying lithium response based on the genomic data of all subjects (ALL; n=321), the poor exemplars (<25th percentile of exemplar score; n=81), and the best exemplars (>75th percentile of exemplar score; n=79). Each panel shows the results for a different classification performance metric. Classification was done using logistic regression with an L2 penalty (regularization weight set to C=1 a priori) with stratification done over each value of the resolution parameter q=1 and q=2. *Abbreviations*: area under the receiver operating characteristic curve (AUC), Cohen's kappa (Kappa), Matthews correlation coefficient (MCC), positive predictive value (PPV), negative predictive value (NPV). Results are presented as means and 95% confidence intervals.

	$q = 1$		$q = 2$		
**Statistic**	**Best**	**Poor**	**Best**	**Poor**	**ALL**
**Accuracy**	0.75 [0.66,0.87]	0.65 [0.53,0.75]	0.75 [0.65,0.75]	0.50 [0.50,0.72]	0.66 [0.60,0.70]
**AUC**	0.88 [0.83,0.98]	0.66 [0.61,0.80]	0.81 [0.66,0.86]	0.53 [0.45,0.72]	0.70 [0.62,0.75]
**Sensitivity**	0.75 [0.50,0.94]	0.50 [0.31,0.75]	0.75 [0.54,0.75]	0.50 [0.06,0.50]	0.59 [0.48,0.62]
**Specificity**	0.88 [0.75,1.]	0.75 [0.75,0.79]	0.75 [0.56,0.94]	0.75 [0.50,1.]	0.70 [0.59,0.83]
**PPV**	0.90 [0.75,1.]	0.67 [0.53,0.75]	0.75 [0.67,0.95]	0.50 [0.12,0.90]	0.67 [0.59,0.78]
**NPV**	0.71 [0.67,0.95]	0.67 [0.53,0.73]	0.75 [0.67,0.79]	0.50 [0.50,0.67]	0.65 [0.61,0.67]
**F1**	0.71 [0.67,0.86]	0.62 [0.39,0.73]	0.71 [0.67,0.79]	0.50 [0.08,0.67]	0.64 [0.58,0.67]
**Kappa**	0.50 [0.31,0.74]	0.28 [0.06,0.50]	0.50 [0.29,0.50]	0. [0.00,0.44]	0.31 [0.20,0.39]
**MCC**	0.58 [0.41,0.77]	0.29 [0.06,0.50]	0.50 [0.39,0.58]	0. [0.00,0.50]	0.32 [0.20,0.44]

2.6 Sensitivity Analyses on Genomic Classification

2.6.1 Preliminaries

Out-of-sample model criticism requires splitting a dataset into training and testing partitions. This is often done repeatedly using cross-validation. Performance estimates will have a higher variance when computed based on smaller test sets. This can easily be shown in closed form as follows. Let N_T be the size of the test set, and N_C the number of examples correctly classified. The probability distribution over N_C is binomial with parameters N_T and $0 < \theta < 1$, where θ is the underlying accuracy of the model. Since the conjugate prior for a binomial likelihood is $\mathrm{Beta}(\theta|\alpha, \beta)$ with hyperparameters (pseudo-counts) $\alpha > 0$ and $\beta > 0$, then the posterior over θ is $\mathrm{Beta}(\theta|\alpha + N_C, \beta + N_T - N_C)$. The posterior variance is

$$\mathrm{Var}(\theta) = \frac{(\alpha + N_C)(\beta - N_C + N_T)}{(\alpha + \beta + N_T)^2 (\alpha + \beta + N_T + 1)}. \tag{6}$$

Under a uniform prior, $\mathrm{Beta}(\theta|\alpha = 1, \beta = 1)$, the maximum likelihood estimate (MLE) of accuracy for a given test set is $\hat{\theta} = N_C/N_T$, and the posterior variance can be rewritten as

$$\mathrm{Var}(\theta) = \frac{-(\hat{\theta} - 1)\hat{\theta} N_T^2 + N_T + 1}{(N_T + 2)^2 (N_T + 3)}. \tag{7}$$

It is trivial to show that Equation 7 is a strictly non-increasing function with respect to N_T, and that its limit in large N_T is zero. It is therefore clear that with small N_T, there is a greater probability of obtaining extreme accuracies (both high and low, as suggested and shown by (9–11)). However, given publication bias, one would consequently expect to see the phenomenon highlighted by Schnack & Kahn (12), whereby larger test set sizes are negatively associated with classification performance. This can be appreciated by visualizing the inverse survival function of the upper tail of $\mathrm{Beta}(\theta|\alpha + N_C, \beta + N_T - N_C)$ under a uniform prior and an MLE of $\hat{\theta}_o = 0.5 = N_C/N_T$, allowing us to substitute $(\alpha + N_C) = (\beta + N_T - N_C) = 1 + N_T/2$. The inverse survival function of this beta distribution, denoted $\psi_o(p, N_T)$, is the value of θ such that $\mathrm{Prob}(X > \theta) = p$ for $0 < X < 1$:

$$\psi_o(p, N_T) = I^{-1}_{(1,-p)}\left(1 + \frac{N_T}{2}, 1 + \frac{N_T}{2}\right), \tag{8}$$

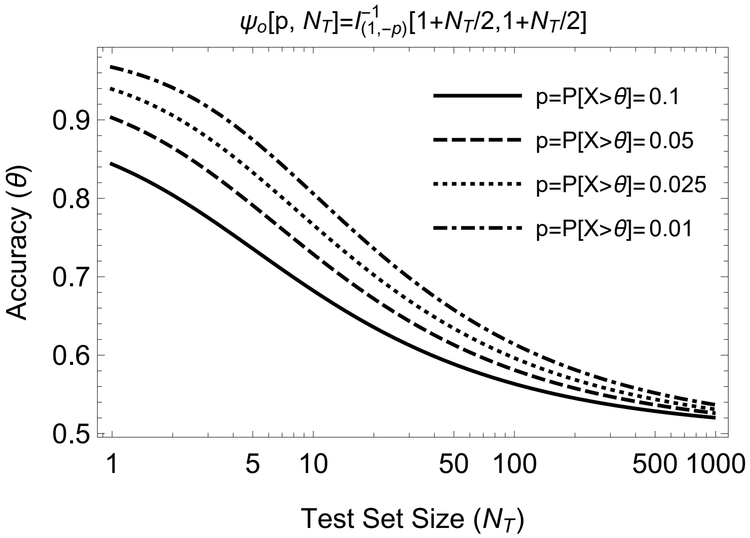

Supplementary Figure 6: The effect of test set size (N_T) on the top $100p^{\text{th}}$ percentile of accuracy expected from a null classifier whose underlying accuracy is actually $\hat{\theta}_o = 0.5$.

where $I^{-1}_{(1,-p)}$ is the inverse of the regularized incomplete beta function. Figure 6 illustrates the effect of test set sample size on top $100p^{\text{th}}$ percentile of accuracy achieved by a "null" or "trivial" classifier whose true underlying accuracy is $\hat{\theta}_o = 0.5$.

Let $\bar{\theta}(N_T)$ be the expected accuracy of a classifier applied to some data, evaluated out-of-sample with test-set size N_T. Our classifier is better than the null if $\text{Prob}(X > \bar{\theta}(N_T))$ is small, or equivalently if $\bar{\theta}(N_T) > \psi_o(p, N_T)$ consistently with respect to N_T for some small value of p. For example, if $\bar{\theta}(N_T = 10) > \psi_o(p = 0.05, N_T = 10)$, then our classifier performs better than 95% of expected null classifiers at a test-set size of 10. Similarly, if $\bar{\theta}(N_T = 10) > \psi_o(p = 0.01, N_T = 10)$, then our classifier has passed an even more stringent test, with better performance than 99% of expected null classifiers.

2.6.2 Sensitivity to Test-Set Size

We repeated our genomic classification experiment for the aggregate sample (denoted "ALL"), as well as the best and poor clinical exemplar strata (those individuals with the top and bottom 25% of exemplar scores, respectively). However, rather than using 10-fold stratified cross-validation, as in the main text, we conducted 100 randomized train-test splits at each of the following test set proportions: $p_{test} \in \{0.1, 0.2, 0.3, 0.4, 0.5, 0.6, 0.7, 0.8\}$. For example, at $p_{test} = 0.1$, we hold out 10% of observations as a test set within the shuffle-split regime.

The classification performance attained at each of the 100 train-test splits conducted at each test-set size N_T (within each of the ALL, best clinical exemplars, and poor clinical exemplars) can be compared to the corresponding value of $\psi_o(p, N_T)$. Figure 7a plots these comparisons across test-set sizes and strata for $p \in \{0.05, 0.025\}$. Figure 7b plots the proportion of shuffle-split runs for which classification performance exceeds $\psi_o(p = 0.05, N_T)$ at each test-set size (expressed as a proportion). One can appreciate that the mean classification accuracy for the poor exemplar stratum never exceeded $\psi_o(p = 0.05, N_T)$. Genomic classification accuracy within the best clinical exemplars was consistently better than that expected from a null model, with the mean classification accuracy exceeding $\psi_o(p = 0.05, N_T)$ at all test-set sizes. Mean classification accuracy within the "ALL" stratum also exceeded $\psi_o(p = 0.05, N_T)$ at test-set proportions $p_{test} > 0.1$.

Figure 8 shows the effects of increasing test-set size on classification performance statistics. The most important finding is that classification performance within the best clinical exemplar stratum was superior to classification within either the whole genomic sample or the poor clinical exemplars. This verifies the central claim of our paper. The area under the receiver operating characteristic curve (AUC) remains on the order of ≥ 0.8 until the test set is increased to $\geq 50\%$ of the total sample size of the best clinical exemplar stratum.

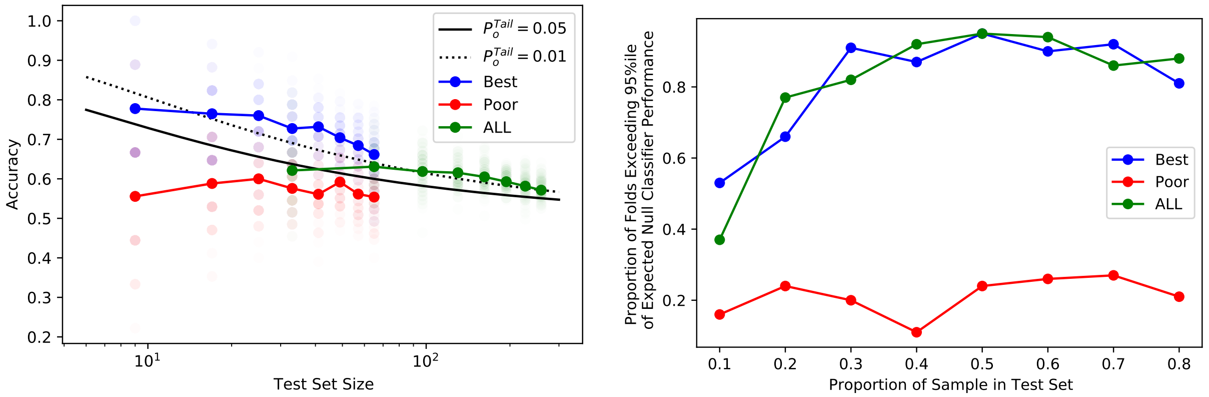

(a) Comparison of classification accuracy to null classifier.

(b) Proportion of classification runs with accuracy exceeding $\psi_o(p = 0.05, N_T)$ at each test-set size.

Supplementary Figure 7: Results of experiment testing the effect of test-set size on genomic classification performance using 100-fold shuffle split cross-validation. **Panel A** plots the performance of genomic classification runs for the best and poor clinical exemplar strata (blue and red points, respectively) and the full sample (ALL; green points). Within each of these strata, we performed 100-fold shuffle split cross validation with test set proportions $p_{test} \in \{0.1, 0.2, 0.3, 0.4, 0.5, 0.6, 0.7, 0.8\}$. The out-of-sample classification accuracy obtained at each run is plotted as a transparent point, coloured by the respective sample stratum. The absolute sample size (on a log scale) is plotted along the x-axis. The solid black line represents the 95th percentile of accuracy ($\psi_o(p = 0.05, N_T)$) that would be expected from a null classifier (i.e. one with accuracy $\hat{\theta}_o = 0.5$). The dotted black line is the 99th percentile of accuracy ($\psi_o(p = 0.01, N_T)$) that would be expected from a null classifier. The solid blue, red, and green lines (with corresponding markers) represent the median classification accuracy for respective strata at each test set size. **Panel B** plots the proportion of classification runs where accuracy exceeded $\psi_o(p = 0.05, N_T)$ for each stratum (best and poor clinical exemplars, and ALL), at each value of p_{test}.

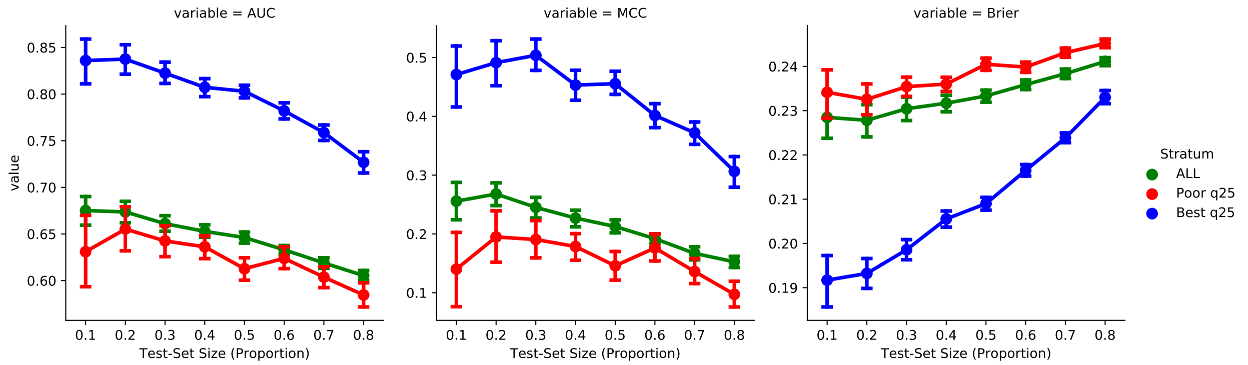

Supplementary Figure 8: Effects of test-set size (expressed as a proportion of total stratum sample size, $p_{test} \in \{0.1, 0.2, 0.3, 0.4, 0.5, 0.6, 0.7, 0.8\}$) on our two primary classification performance statistics (area under the receiver operating characteristic curve [AUC], and Matthews correlation coefficient [MCC]). We have additionally included the Brier score. Points are means and error bars are 95% confidence intervals computed across 100 iterations of shuffle-split cross-validation.

2.7 Results of Gene Enrichment Analysis

Results of the pathway analysis are shown in Table 4. Genes that were enriched among the glutamatergic synapse cellular component are shown in Table 5. Genes that were enriched among the glutamatergic signalling biological process are shown in Table 6.

Supplementary Table 4: Results of gene enrichment analysis using the PANTHER gene ontology system. Analyses are presented for (A) pathways, (B) gene ontology cellular components, and (C) gene ontology biological processes. *Abbreviations*: false discovery rate (FDR).

	Best				Poor			
	**N**	**+/-**	**P value**	**FDR**	**N**	**+/-**	**P value**	**FDR**
Pathways								
Muscarinic acetylcholine receptor 1 and 3 signaling pathway	27	+	0.00011	0.017				
Alzheimer disease-amyloid secretase pathway	30	+	0.00045	0.034				
Heterotrimeric G-protein signaling pathway-Gq alpha and Go alpha mediated pathway	53	+	0.00081	0.041				
Histamine H1 receptor mediated signaling pathway	27	+	0.00103	0.039				
Cellular component								
glutamatergic synapse (GO:0098978)	159	+	6.02E-08	3.05E-05				
synapse (GO:0045202)	468	+	3.90E-09	5.93E-06				
neuron projection (GO:0043005)	489	+	6.68E-05	7.25E-03				
neuron part (GO:0097458)	657	+	7.12E-07	1.08E-04				
cation channel complex (GO:0034703)					109	+	6.01E-05	7.10E-03
ion channel complex (GO:0034702)					139	+	1.09E-04	9.83E-03
transmembrane transporter complex (GO:1902495)					145	+	2.54E-04	1.70E-02
transporter complex (GO:1990351)					146	+	3.88E-04	2.48E-02
ionotropic glutamate receptor complex (GO:0008328)	28	+	1.20E-04	1.07E-02	27	+	1.32E-04	1.07E-02
plasma membrane part (GO:0044459)	1041	+	7.13E-04	4.52E-02				
neurotransmitter receptor complex (GO:0098878)	28	+	1.20E-04	1.14E-02	27	+	1.32E-04	1.13E-02
integral component of postsynaptic density membrane (GO:0099061)	35	+	3.16E-04	2.40E-02	33	+	6.62E-05	7.26E-03
intrinsic component of postsynaptic density membrane (GO:0099146)	36	+	1.95E-04	1.64E-02	34	+	1.37E-04	1.05E-02
intrinsic component of postsynaptic specialization membrane (GO:0098948)					39	+	5.09E-05	6.51E-03
intrinsic component of postsynaptic membrane (GO:0098936)	66	+	4.85E-04	3.35E-02	62	+	3.72E-05	5.71E-03
intrinsic component of synaptic membrane (GO:0099240)	91	+	6.09E-05	7.72E-03	85	+	1.24E-05	3.18E-03
synapse part (GO:0044456)	368	+	5.20E-07	9.87E-05	374	+	3.33E-05	5.68E-03
synapse (GO:0045202)					469	+	7.61E-07	3.89E-04
synaptic membrane (GO:0097060)	202	+	2.23E-08	1.70E-05	203	+	3.30E-07	2.53E-04
postsynaptic membrane (GO:0045211)	150	+	7.48E-08	2.84E-05	154	+	2.33E-07	3.58E-04
postsynapse (GO:0098794)	243	+	1.50E-07	4.56E-05	250	+	4.97E-04	2.93E-02
postsynaptic specialization membrane (GO:0099634)	54	+	4.22E-04	3.05E-02	50	+	3.99E-05	5.57E-03
postsynaptic specialization (GO:0099572)	146	+	1.59E-06	2.20E-04				
neuron part (GO:0097458)					662	+	1.38E-06	5.28E-04
postsynaptic density membrane (GO:0098839)	45	+	6.17E-05	7.22E-03	44	+	1.05E-04	1.00E-02
postsynaptic density (GO:0014069)	140	+	5.32E-07	8.99E-05				
asymmetric synapse (GO:0032279)	142	+	1.77E-07	4.49E-05				
neuron to neuron synapse (GO:0098984)	149	+	2.67E-07	5.81E-05				
integral component of postsynaptic specialization membrane (GO:0099060)					38	+	2.49E-05	4.78E-03
integral component of postsynaptic membrane (GO:0099055)	63	+	2.76E-04	2.21E-02	59	+	1.32E-05	2.89E-03
integral component of synaptic membrane (GO:0099699)	84	+	1.05E-04	1.07E-02	78	+	1.01E-05	3.10E-03
cell junction (GO:0030054)	478	+	6.31E-04	4.17E-02	478	+	9.19E-05	9.40E-03
neuron projection (GO:0043005)					501	+	1.73E-04	1.26E-02
presynaptic membrane (GO:0042734)					80	+	3.97E-04	2.44E-02
presynapse (GO:0098793)					196	+	2.46E-04	1.72E-02
Biological process								
regulation of cell morphogenesis involved in differentiation (GO:0010769)	116	+	1.09E-05	2.57E-02				
regulation of cell morphogenesis (GO:0022604)	189	+	2.31E-05	2.28E-02				
synapse organization (GO:0050808)	114	+	1.70E-05	2.87E-02				
axon guidance (GO:0007411)	121	+	1.75E-05	2.58E-02				
cell development (GO:0048468)	595	+	2.96E-05	2.50E-02				
cell differentiation (GO:0030154)	1174	+	8.13E-05	4.80E-02				
developmental process (GO:0032502)	1819	+	6.38E-05	4.19E-02				

Continued on next page...

Supplementary Table 4: Continued

	Best				Poor			
	**N**	**+/-**	**P value**	**FDR**	**N**	**+/-**	**P value**	**FDR**
anatomical structure development (GO:0048856)	1742	+	4.46E-05	3.51E-02				
generation of neurons (GO:0048699)	551	+	5.13E-05	3.79E-02				
neurogenesis (GO:0022008)	583	+	1.86E-05	2.19E-02				
nervous system development (GO:0007399)	819	+	6.40E-06	3.78E-02				
system development (GO:0048731)	1462	+	3.69E-06	4.35E-02				
multicellular organism development (GO:0007275)	1631	+	6.43E-05	3.99E-02				
neuron projection guidance (GO:0097485)	123	+	1.93E-05	2.07E-02				
regulation of neuron projection development (GO:0010975)	190	+	1.79E-05	2.35E-02				
regulation of neuron differentiation (GO:0045664)	243	+	9.89E-06	3.89E-02				
regulation of plasma membrane bounded cell projection organization (GO:0120035)	249	+	1.02E-05	3.00E-02				
regulation of cell projection organization (GO:0031344)	250	+	1.40E-05	2.76E-02				
glutamate receptor signaling pathway (GO:0007215)	30	+	2.49E-05	2.26E-02				
circulatory system development (GO:0072359)	314	+	5.58E-05	3.88E-02				
modulation of chemical synaptic transmission (GO:0050804)					182	+	4.51E-06	5.32E-02
regulation of trans-synaptic signaling (GO:0099177)					182	+	4.51E-06	2.66E-02
cell-cell adhesion via plasma-membrane adhesion molecules (GO:0098742)					99	+	9.19E-06	3.62E-02

Supplementary Table 5: Genes enriched in the best exemplars group related to glutamatergic synapses (gene ontology “cellular component” category).

**Gene**	**Gene Symbol**	**Protein Class**
ABR	Active breakpoint cluster region-related protein	guanyl-nucleotide exchange factor(PC00113)
ACAN	Aggrecan core protein	extracellular matrix glycoprotein(PC00100)
ACTN1, ACTN2	Alpha-actinin-1 & 2	
ADAM22, ADAM23	Disintegrin and metalloproteinase domain-containing protein 22 & 23	metalloprotease(PC00153)
ADCY1, ADCY8	Adenylate cyclase type 1 & 8	
ADGRL3	Adhesion G protein-coupled receptor L3	G-protein coupled receptor(PC00021), antibacterial response protein(PC00051), protease(PC00190)
ADORA2B	Adenosine receptor A2b	G-protein coupled receptor(PC00021)
ADRA1A	Alpha-1A adrenergic receptor	G-protein coupled receptor(PC00021)
APBA1	Amyloid-beta A4 precursor protein-binding family A member 1	membrane trafficking regulatory protein(PC00151)
ARHGAP22, ARHGAP39, ARHGAP44	Rho GTPase-activating protein 22	
ATP2B2, ATP2B4	Plasma membrane calcium-transporting ATPase 2 & 4	cation transporter(PC00068), hydrolase(PC00121), ion channel(PC00133)
BAIAP2	Brain-specific angiogenesis inhibitor 1-associated protein 2	receptor(PC00197)
BCR	Breakpoint cluster region protein	guanyl-nucleotide exchange factor(PC00113)
CACNA1A	Voltage-dependent P/Q-type calcium channel subunit alpha-1A	
CACNG2, CACNG3, CACNG4	Voltage-dependent calcium channel gamma-2 subunit	voltage-gated calcium channel(PC00240)
CADPS, CADPS2	Calcium-dependent secretion activator 1 & 2	calcium-binding protein(PC00060)
CAMK4	Calcium/calmodulin-dependent protein kinase type IV	non-motor microtubule binding protein(PC00166), non-receptor serine/threonine protein kinase(PC00167)
CDH8, CDH10, CDH11	Cadherin-8,10,11	
CHMP2B	Charged multivesicular body protein 2b	
CHRM2, CHRM3	Muscarinic acetylcholine receptor M2 & M3	G-protein coupled receptor(PC00021)
CLSTN1, CLSTN2	Calsyntenin-1 & 2	calcium-binding protein(PC00060), cell adhesion molecule(PC00069)
CNR1	Cannabinoid receptor 1	G-protein coupled receptor(PC00021)
CPLX2	Complexin-2	
CTBP2	C-terminal-binding protein 2	transcription cofactor(PC00217)
CTTNBP2	Cortactin-binding protein 2	
DGKB	Diacylglycerol kinase beta	kinase(PC00137)
DGKI	Diacylglycerol kinase iota	kinase(PC00137)
DLG2	Disks large homolog 2	transmembrane receptor regulatory/adaptor protein(PC00226)

Continued on next page...

Supplementary Table 5: Continued

Gene	Gene Symbol	Protein Class
DLGAP4	Disks large-associated protein 4	transmembrane receptor regulatory/adaptor protein(PC00226)
DNM2, DNM3	Dynamin-2 & 3	hydrolase(PC00121), microtubule family cytoskeletal protein(PC00157), small GTPase(PC00208)
DRD2, DRD3	D(2) & D(3) dopamine receptors	G-protein coupled receptor(PC00021)
EFNB2	Ephrin-B2	membrane-bound signaling molecule(PC00152)
EPHA4, EPHA7	Ephrin type-A receptors 4 & 7	
EPHB1, EPHB2	Ephrin type-B receptors 1 & 2	
ERBB4	Receptor tyrosine-protein kinase erbB-4	
ERC2	ERC protein 2	G-protein modulator(PC00022), membrane traffic protein(PC00150)
FARP1	FERM, ARHGEF and pleckstrin domain-containing protein 1	
FYN	Tyrosine-protein kinase Fyn	
FZD3	Frizzled-9	G-protein coupled receptor(PC00021), protease inhibitor(PC00191), signaling molecule(PC00207)
GABRR1	Gamma-aminobutyric acid receptor subunit rho-1	GABA receptor(PC00023), acetylcholine receptor(PC00037)
GPC6	Glypican-6	
GPM6A	Neuronal membrane glycoprotein M6-a	myelin protein(PC00161)
GRIA1	Glutamate receptor 1	
GRID1, GRID2	Glutamate receptor ionotropic, delta-1 & 2	
GRIK2, GRIK5	Glutamate receptor ionotropic, kainate 2 & 5	
GRIN2A, GRIN3A	Glutamate receptor ionotropic, NMDA 2A & 3A	
GRIP1, GRIP2	Glutamate receptor-interacting protein 1 & 2	
GRM1, GRM3	Metabotropic glutamate receptor 1 & 3	G-protein coupled receptor(PC00021)
GSG1L	Germ cell-specific gene 1-like protein	cytoskeletal protein(PC00085)
GSK3B	Glycogen synthase kinase-3 beta	non-receptor serine/threonine protein kinase(PC00167)
HIP1	Huntingtin-interacting protein 1	non-motor actin binding protein(PC00165)
HOMER1, HOMER2	Homer protein homolog 1 & 2	
HTR2A	5-hydroxytryptamine receptor 2A	G-protein coupled receptor(PC00021)
IL1RAP	Interleukin-1 receptor accessory protein	type I cytokine receptor(PC00231)
ITGB1, ITGB3	Integrin beta-1 & 3	cell adhesion molecule(PC00069), receptor(PC00197)
ITSN1	Intersectin-1	G-protein modulator(PC00022);calcium-binding protein(PC00060);membrane traffic protein(PC00150)
KCND2	Potassium voltage-gated channel subfamily D member 2	

Continued on next page...

Supplementary Table 5: Continued

Gene	Gene Symbol	Protein Class
LGI1	Leucine-rich glioma-inactivated protein 1	
LRFN5	Leucine-rich repeat and fibronectin type-III domain-containing protein 5	
LRRC4C	Leucine-rich repeat-containing protein 4C	
LRRK2	Leucine-rich repeat serine/threonine-protein kinase 2	
LRRN2	Leucine-rich repeat transmembrane neuronal protein 2	
LRRTM4	Leucine-rich repeat transmembrane neuronal protein 4	extracellular matrix protein(PC00102), receptor(PC00197)
LYN	Tyrosine-protein kinase Lyn	
MAPK10, MAPK14	Mitogen-activated protein kinase 10 & 14	non-receptor serine/threonine protein kinase(PC00167)
MTOR	Serine/threonine-protein kinase mTOR	non-receptor serine/threonine protein kinase(PC00167);nucleic acid binding(PC00171);nucleotide kinase(PC00172)
NAPB	Beta-soluble NSF attachment protein	membrane traffic protein(PC00150)
NDRG1	Protein NDRG1	serine protease(PC00203)
NETO1	Neuropilin and tolloid-like protein 1	
NLGN1	Neuroligin-1	
NOS1AP	Carboxyl-terminal PDZ ligand of neuronal nitric oxide synthase protein	signaling molecule(PC00207)
NRCAM	Neuronal cell adhesion molecule	
NRG1, NRG3	Pro-neuregulin-1 & 3, membrane-bound isoform	growth factor(PC00112)
NRP1, NRP2	Neuropilin-1 & 2	
NRXN1	Neurexin-1	
NTNG1, NTNG2	Netrin-G1 & G2	extracellular matrix linker protein(PC00101), protease inhibitor(PC00191), receptor(PC00197)
NTRK3	NT-3 growth factor receptor	
OLFM2	Noelin-2	receptor(PC00197);structural protein(PC00211)
P2RY1	P2Y purinoceptor 1	
PAK2	Serine/threonine-protein kinase PAK 2	
PLCB1, PLCB4	1-phosphatidylinositol 4,5-bisphosphate phosphodiesterase beta-1 & 4	calcium-binding protein(PC00060), guanyl-nucleotide exchange factor(PC00113), phospholipase(PC00186), signaling molecule(PC00207)
PLEKHA5	Pleckstrin homology domain-containing family A member 5	
PLPPR4	Phospholipid phosphatase-related protein type 4	phosphatase(PC00181);pyrophosphatase(PC00196)
PPFIA2	Liprin-alpha-2 & 3	

Continued on next page...

Supplementary Table 5: Continued

Gene	Gene Symbol	Protein Class
PPFIA3		
PPM1H	Protein phosphatase 1H	kinase inhibitor(PC00139), protein phosphatase(PC00195)
PPP1R9A	Neurabin-1	
PPP3CA	Serine/threonine-protein phosphatase 2B catalytic subunit alpha isoform	
PRKAR1A	cAMP-dependent protein kinase type I-alpha regulatory subunit	
PSD2	PH and SEC7 domain-containing protein 2	
PTK2B	Protein-tyrosine kinase 2-beta	
PTPRD	Receptor-type tyrosine-protein phosphatase delta	protein phosphatase(PC00195);receptor(PC00197)
PTPRO, PTPRS, PTPRT	Receptor-type tyrosine-protein phosphatase O, S, & T	protein phosphatase(PC00195)
RAC1	Ras-related C3 botulinum toxin substrate 1	small GTPase(PC00208)
RAP1A	Ras-related protein Rap-1A	small GTPase(PC00208)
RGS7BP	Regulator of G-protein signaling 7-binding protein	
RNF216	E3 ubiquitin-protein ligase RNF216	
SCN2A	Sodium channel protein types 2 & 10 10 subunit alpha	voltage-gated calcium channel(PC00240)
SCN10A		voltage-gated sodium channel(PC00243)
SH3GL1, SHGL2, SHGL3	Endophilin-A2,A1, & A3	
SHANK2	SH3 and multiple ankyrin repeat domains protein 2	
SHISA6, SHISA9	Protein shisa-6 & 9	
SLC1A2, SLC1A6	Excitatory amino acid transporter 2	cation transporter(PC00068)
SLC6A17	Sodium-dependent neutral amino acid transporter SLC6A17	cation transporter(PC00068)
SNAP25	Synaptosomal-associated protein 25	SNARE protein(PC00034)
SORCS3	VPS10 domain-containing receptor SorCS3	receptor(PC00197), transporter(PC00227)
SPARC, SPARCL1	SPARC & SPARC-like protein 1	cell adhesion molecule(PC00069), extracellular matrix glycoprotein(PC00100), growth factor(PC00112)
SPTBN1	Spectrin beta chain, non-erythrocytic 1	
SRC	Proto-oncogene tyrosine-protein kinase Src	
STX3	Syntaxin-3	SNARE protein(PC00034)
SV2A	Synaptic vesicle glycoprotein 2A	
SYN3	Synapsin-3	membrane trafficking regulatory protein(PC00151);non-motor actin binding protein(PC00165)

Continued on next page...

Supplementary Table 5: Continued

Gene	Gene Symbol	Protein Class
SYNPO	Synaptopodin	non-motor actin binding protein(PC00165)
SYT1, SYT6	Synaptotagmin-1 & 6	membrane trafficking regulatory protein(PC00151)
TANC2	Protein TANC2	
TIAM1	T-lymphoma invasion and metastasis-inducing protein 1	
TNIK	TRAF2 and NCK-interacting protein kinase	
TNR	Tenascin-R	signaling molecule(PC00207)
UNC13A	Protein unc-13 homolog A	
WASF3	Wiskott-Aldrich syndrome protein family member 3	non-motor actin binding protein(PC00165)
WNT7A	Protein Wnt-7a	signaling molecule(PC00207)
YWHAZ	14-3-3 protein zeta/delta	chaperone(PC00072)

Supplementary Table 6: Genes enriched among the best exemplars in the gene ontology "biological process" category of the glutamate receptor signaling pathway.

Gene	Gene Symbol	Protein Class
APP	Amyloid-beta A4 protein	protease inhibitor(PC00191)
GNAQ	Guanine nucleotide-binding protein G(q) subunit alpha	heterotrimeric G-protein(PC00117)
GRIA1, GRIA4	Glutamate receptor 1 & 4	
GRID1, GRID2	Glutamate receptor ionotropic, delta-1, 2	
GRIK1, GRIK2, GRIK4, GRIK5	Glutamate receptor ionotropic, kainate 1,2,4,5	
GRIN2A, GRIN2B, GRIN2D, GRIN3A	Glutamate receptor ionotropic, NMDA 2A, 2B, 2D, 3A	
GRM1, GRM3, GRM4, GRM5, GRM6, GRM7, GRM8	Metabotropic glutamate receptor 1,3,4,5,6,7,8	G-protein coupled receptor(PC00021)
HOMER1, HOMER2	Homer protein homolog 1 & 2	
KCNB1	Potassium voltage-gated channel subfamily B member 1	
PLCB1	1-phosphatidylinositol 4,5-bisphosphate phosphodiesterase beta-1	calcium-binding protein(PC00060), guanyl-nucleotide exchange factor(PC00113), phospholipase(PC00186), signaling molecule(PC00207)
PTK2B	Protein-tyrosine kinase 2-beta	
SSR1	Somatostatin receptor type 1	G-protein coupled receptor(PC00021)
TIAM1	T-lymphoma invasion and metastasis-inducing protein 1	
TRPM1, TRPM3	Transient receptor potential cation channel subfamily M member 1 & 3	ion channel(PC00133), receptor(PC00197)

3 Supplementary Discussion

3.1 Further Rationale for SNP Set Used in Classification Analyses

Filtering-based feature selection approaches in our present study would be (A) too computationally expensive across these millions of variants and (B) require much larger sample sizes since they must be repeated within each training partition. We also had no dominant a priori biological rationale for limiting the data to a restricted subset, since, as our results later confirmed, these biological systems may differ between exemplar strata. Ultimately, we chose the set of completely genotyped SNPs that overlapped across ConLiGen sites in order to facilitate the potential conceptual generalizability of our pathway analysis results, in particular. That is, since the pathways detected were based on variants that are broadly genotyped, these results could potentially be extended to other ConLiGen sites, should the corresponding clinical variables become available.

References

[1] Nunes A, Alda M, Bardouille T, Trappenberg T. Representational Rényi heterogeneity *arXiv.* 2019.

[2] Breiman L. Random Forests *Machine Learning.* 2001;45:5–32.

[3] Nunes A, Ardau R, Berghöfer A, et al. Prediction of Lithium Response using Clinical Data *Acta Psychiatrica Scandinavica.* 2019;In Press.

[4] Pedregosa F, Varoquaux G, Gramfort A, et al. Scikit-learn: Machine Learning in Python *Journal of Machine Learning Research.* 2012;12:2825–2830.

[5] Mi Huaiyu, Muruganujan Anushya, Huang Xiaosong, et al. Protocol Update for large-scale genome and gene function analysis with the PANTHER classification system (v.14.0) *Nature Protocols.* 2019;14:703–721.

[6] Hou L, Heilbronner U, Degenhardt F, et al. Genetic variants associated with response to lithium treatment in bipolar disorder: A genome-wide association study *The Lancet.* 2016;387:1085–1093.

[7] Turecki G, Grof P, Cavazzoni P, et al. Evidence for a role of phospholipase C-γ1 in the pathogenesis of bipolar disorder *Molecular Psychiatry.* 1998;3:534–538.

[8] Turecki G, Grof P, Grof E, et al. Mapping susceptibility genes for bipolar disorder: A pharmacogenetic approach based on excellent response to lithium *Molecular Psychiatry.* 2001;6:570–578.

[9] Varoquaux Gaël. Cross-validation failure: Small sample sizes lead to large error bars *NeuroImage.* 2018;180:68–77.

[10] Little Max A., Varoquaux Gael, Saeb Sohrab, et al. Using and understanding cross-validation strategies. Perspectives on Saeb et al *GigaScience.* 2017;6:1–6.

[11] Flint Claas, Cearns Micah, Opel Nils, et al. Systematic Overestimation of Machine Learning Performance in Neuroimaging Studies of Depression 2019.

[12] Schnack HG, Kahn RS. Detecting neuroimaging biomarkers for psychiatric disorders: Sample size matters. *Frontiers in Psychiatry.* 2016;7.
